# An Extended C-Terminus, the Possible Culprit for Differential Regulation of 5-Aminolevulinate Synthase Isoforms

**DOI:** 10.3389/fmolb.2022.920668

**Published:** 2022-07-14

**Authors:** Gregory A. Hunter, Gloria C. Ferreira

**Affiliations:** ^1^ Department of Molecular Medicine, Morsani College of Medicine, University of South Florida, Tampa, FL, United States; ^2^ Department of Chemistry, College of Arts and Sciences, University of South Florida, Tampa, FL, United States; ^3^ Global and Planetary Health, College of Public Health, University of South Florida, Tampa, FL, United States

**Keywords:** 5-aminolevulinate synthase, pyridoxal 5′-phosphate, heme regulatory motif, allostery, redox sensor, porphyrin, regulation, AlphaFold

## Abstract

5-Aminolevulinate synthase (ALAS; E.C. 2.3.1.37) is a pyridoxal 5′-phosphate (PLP)-dependent enzyme that catalyzes the key regulatory step of porphyrin biosynthesis in metazoa, fungi, and α-proteobacteria. ALAS is evolutionarily related to transaminases and is therefore classified as a fold type I PLP-dependent enzyme. As an enzyme controlling the key committed and rate-determining step of a crucial biochemical pathway ALAS is ideally positioned to be subject to allosteric feedback inhibition. Extensive kinetic and mutational studies demonstrated that the overall enzyme reaction is limited by subtle conformational changes of a hairpin loop gating the active site. These findings, coupled with structural information, facilitated early prediction of allosteric regulation of activity via an extended C-terminal tail unique to eukaryotic forms of the enzyme. This prediction was subsequently supported by the discoveries that mutations in the extended C-terminus of the erythroid ALAS isoform (ALAS2) cause a metabolic disorder known as X-linked protoporphyria not by diminishing activity, but by enhancing it. Furthermore, kinetic, structural, and molecular modeling studies demonstrated that the extended C-terminal tail controls the catalytic rate by modulating conformational flexibility of the active site loop. However, the precise identity of any such molecule remains to be defined. Here we discuss the most plausible allosteric regulators of ALAS activity based on divergences in AlphaFold-predicted ALAS structures and suggest how the mystery of the mechanism whereby the extended C-terminus of mammalian ALASs allosterically controls the rate of porphyrin biosynthesis might be unraveled.

## Introduction

5-Aminolevulinate synthase (ALAS; EC 2.3.1.37) catalyzes the initial and key regulatory step of heme biosynthesis in metazoa, fungi, and the α-subclass of proteobacteria (Stojanovski et al., 2019; Taylor and Brown, 2022). Pyridoxal 5′-phosphate (PLP) is an essential cofactor for the reaction, which involves the condensation of the α-carbon of glycine with the succinyl group of succinyl-Coenzyme A (SCoA) to produce 5-aminolevulinate (ALA), carbon dioxide, and Coenzyme A (Hunter and Ferreira, 2011) (Supplementary Figure S1). In metazoa and fungi, ALAS is translated as a precursor with an N-terminal signal sequence that codes for import into the mitochondrial matrix. Following import, the signal sequence is cleaved, and the mature enzyme has access to the substrate SCoA, which is produced in mitochondria as part of the citric acid cycle. The requirement of SCoA as a substrate integrates heme biosynthesis with oxidative respiration, and as a result the two pathways are synchronized under normal healthy conditions. ALAS activity is additionally synchronized with cellular iron transport as porphyrin biosynthesis and iron transport unite in the final step of heme production wherein the enzyme ferrochelatase inserts ferrous iron into protoporphyrin IX to yield heme (Kafina and Paw, 2017; Poli et al., 2021). As a result of the central position of ALAS in these fundamental biochemical pathways ALAS activity is highly regulated and new modes of ALAS regulation continue to be discovered (Tanimura et al., 2016; Zhang et al., 2017; Liu et al., 2018; Peoc'h et al., 2019; Bailey et al., 2020; Nomura et al., 2021; Rondelli et al., 2021).

**FIGURE 1:**
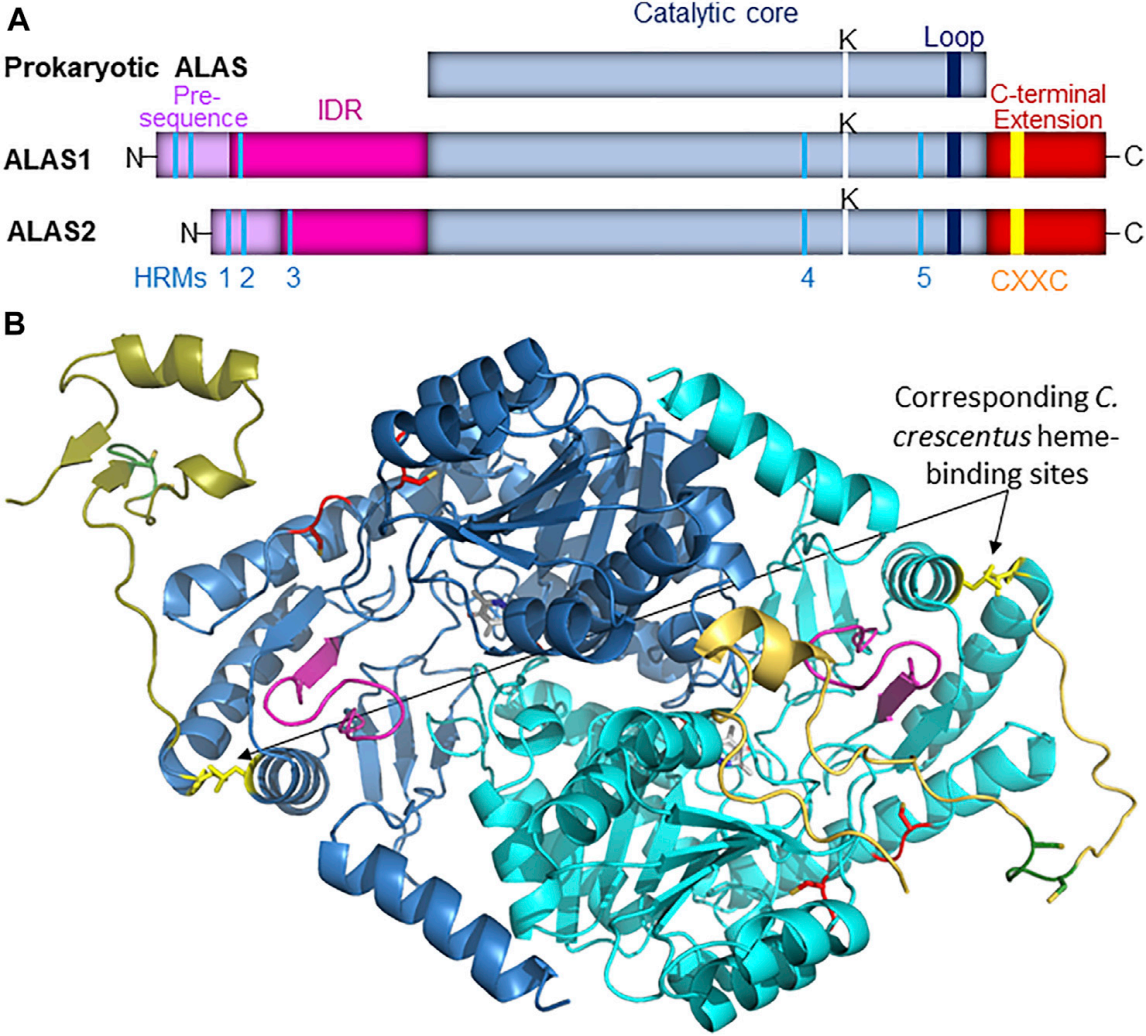
**(A)**. Schematic representation of ALAS monomeric structure. In vertebrate species the ALAS gene is duplicated, and the protein catalytic core (light blue) observed in prokaryotes is bracketed by extended N- and C-termini. The mitochondrial targeting sequence is illustrated in light purple, while the intrinsically disordered N-terminal extension (IDR) is in magenta, and the C-terminal extension is in dark red. Five conserved Heme Regulatory Motifs (HRMs, colored in cyan) are conserved in vertebrate ALAS isozymes, as is a CXXC motif (yellow) in the extended C-terminus. The position of the active site lysine residue that binds PLP in the active site is denoted by a white line, and the loop that gates the active site is represented by a dark blue line. **(B)**. The position corresponding to the heme-binding site in *C. crescentus* ALAS modeled into mammalian ALAS2. AlphaFold-predicted structures for human (light blue and gold; AlphaFold entry P22557) and murine (dark blue and gold; AlphaFold entry P08680) ALAS2 were aligned with *R. capsulatus* ALAS crystal structure (PDB code 2bwn; not shown) using Pymol. The modeled site depicted in yellow here is not expected to bind heme in mammals due to evolutionary divergences, and this site is illustrated solely for perspective on its spatial relationship to the mammalian ALAS2 active site loop (purple), the C-terminal extension (shades of gold), and HRMs 4 and 5 (red). Additionally, the CXXC motifs are in green with the cysteines shown as sticks.

Vertebrate genomes encode two chromosomally distinct copies of the ALAS gene: *ALAS1*, which acts as a “housekeeping” gene and initiates heme biosynthesis in all cells for production of cytochromes and other heme-binding proteins, and *ALAS2*, which is expressed only in developing erythrocytes and produces, almost exclusively, the much larger quantities of heme required for hemoglobin formation (Riddle et al., 1989; Peoc'h et al., 2019). The catalytic cores of human ALAS1 and ALAS2 are 75% identical and 94% similar in terms of amino acid sequences, suggesting gene duplication and similar enzymology despite the different metabolic functionalities of the gene products. The high degree of similarity in the catalytic cores of ALAS1 and ALAS2 is lessened in the extended N- and C-termini of the enzymes (Supplementary Figure S2) but the precise extent to which the mature mitochondrial enzymes might be differentially regulated is still an open question. The monomeric primary structures of prokaryotic and vertebrate ALASs are illustrated schematically in Figure 1A.

The ALAS-catalyzed reaction not only represents the first committed step of heme production, but also the rate-determining step of porphyrin biosynthesis, as most poignantly evinced by the consistent observation that exogenous ALA administration to mammalian cells leads to rapid protoporphyrin IX accumulation (Hunter and Ferreira, 2011; Nokes et al., 2013). This is clinically important because it means aberrations in ALAS activity can change the overall rate of porphyrin production and cause porphyrin biosynthesis to decouple from oxidative respiration and iron transport, resulting in metabolic imbalances (Taylor and Brown, 2022). For instance, certain liver toxins, such as allylisopropylacetamide, have long been known to elevate ALAS1 activity beyond the rate of iron transport, resulting in porphyrin accumulation and chemically induced porphyria (Goldberg and Rimington, 1955; Granick, 1966). Conversely, genetic defects in ALAS2 that lead to lower enzymatic activity have been identified as the basis for X-linked sideroblastic anemia, a condition characterized by accumulation of iron in erythroblast mitochondria (Abu-Zeinah and DeSancho, 2020). Remarkably, however, loss-of-function mutations are not the only cause of ALAS2-associated metabolic disorder. A limited number of mutations causing premature truncation or frameshifts in the extreme C-terminal extension of ALAS2 lead to variants with increased catalytic efficiencies and a disorder known as X-linked protoporphyria (Whatley et al., 2008; Ducamp et al., 2013; Wang et al., 2020). Interestingly, mutations in ALAS1 have not been associated with any disorder (Stenson et al., 2003).

## 5-Aminolevulinate Synthase is a Fold Type I Pyridoxal 5′-Phosphate-dependent Enzyme With a Distinct Active Site Loop

PLP-dependent enzymes are structurally classified into seven different fold types, of which fold type I, sometimes referred to as the transaminase family, is by far the largest, with over 170 different Enzyme Classification numbers currently assigned (Percudani and Peracchi, 2009). Like other members of the PLP-dependent fold type I family ALAS is a homodimer with the active site buried near the center of the enzyme at the interface between the two monomers, with residues from each monomer being critical for substrate recognition (Brown et al., 2018; Stojanovski et al., 2019). Even though fold type I PLP-dependent enzymes have very little overall primary sequence similarity the active sites are highly conserved and facilitate phylogenetic analyses demonstrating function-based evolutionary relationships (Catazaro et al., 2014). It is thus informative to compare the structure of aspartate aminotransferase (AATase), which has been extensively characterized and is generally considered to be a model for the fold type I family (Toney, 2014), with the ALAS catalytic core, as seen in Supplementary Figure S3. The aligned structures of AATase in the open and closed conformations reveal the structure collapses inwards towards the PLP cofactor upon substrate binding (McPhalen et al., 1992a; McPhalen et al., 1992b) A short active site loop (green and gold in Supplementary Figure S3A) closes inward over the active site cleft upon substrate binding, culminating in an arginine residue that is highly conserved in fold type I enzymes, and functions to form an ionic bond with the carboxylate group of the amino acid substrate (Tan et al., 1998; Liang et al., 2019). In AATase, this arginine is one of only two amino acids that has been designated as a “closure-inducing residue”, meaning it is essential for substrate-induced conformational change from the open to the closed state in which catalysis is optimized (Hayward, 2004). Comparison of these structures to analogous structures of *Rhodobacter capsulatus* ALAS (Supplementary Figure S3B) reveals that in ALAS substrate-induced conformational changes are largely limited to the active site loop, which has become longer and is turned more inward over the active site cleft relative to AATase.

Detailed mutational, kinetic, and molecular modeling studies have found that the rate of ALAS catalysis, and hence the rate of porphyrin production, are controlled by the slow opening of this active site loop, which allows the products to rapidly dissociate from the enzyme (Hunter and Ferreira, 1999; Hunter and Ferreira, 2011; Hunter et al., 2007; Stojanovski et al., 2019). This rate-dependence on conformational dynamics would seem to be an ideal situation for allosteric feedback inhibition of the heme biosynthesis pathway via a mechanism wherein effector binding to ALAS would modulate the active site loop conformational dynamics, as we previously suggested (Hunter et al., 2007).

## 5-Aminolevulinate Synthase Structural Features Reveal important Clues to the Possibility of Allosteric Regulation

Feedback inhibition of ALAS activity by heme has been known for over 50 years (Granick, 1966), and since then this regulation has been found to occur at a variety of levels, including gene transcription (Yamamoto et al., 1982), transport into mitochondria (Lathrop and Timko, 1993; Munakata et al., 2004), and targeting for degradation (Cable et al., 1996; Yoshino et al., 2007; Tian et al., 2011; Nomura et al., 2021). However, as of this writing direct binding of heme leading to allosteric feedback inhibition of ALAS has only been reported for the enzyme from the prokaryote *Caulobacter crescentus*, in which axial heme binding by H340 and C398 near the C-terminus of the enzyme causes PLP dissociation (Ikushiro et al., 2018) (Figure 1B). While the authors reported that these residues are conserved in some other α-proteobacteria and did confirm that recombinant *R. capsulatus* ALAS could also be isolated as a mixture of PLP- and heme-bound forms, these residues are not conserved in eukaryotes, so if allosteric feedback inhibition of ALAS in higher species occurs it must be *via* a different site. The recently resolved crystal structure for human ALAS2 revealed that the extended C-terminus might act as an autoinhibitory element by folding directly over the active site cleft, clearly implying the existence of some allosteric modulator that alters the conformational dynamics about the C-terminus to allow substrates to access the active site (Bailey et al., 2020), and yet the identity of this effector remains a mystery.

Each of the vertebrate ALAS isozymes contains five heme-regulatory motifs (HRMs), consensus sequences containing a cysteine-proline dipeptide with the cysteine functioning as a ligand to Fe^3+^-heme (Figure 1A) (Carter et al., 2017; Fleischhacker et al., 2020). HRMs are important in regulating the activity of a wide variety of enzymes controlling gene transcription (Hou et al., 2006; Fleischhacker et al., 2018; Arunachalam et al., 2021), protein synthesis (Igarashi et al., 2008), circadian rhythms (Yang et al., 2008), iron homeostasis (Nishitani et al., 2019), signal transduction (Shen et al., 2014; Schmalohr et al., 2021), and heme degradation (Fleischhacker et al., 2015; Fleischhacker et al., 2018). The first two ALAS HRMs reside in the mitochondrial import signal sequence, where they are positioned to bind excess labile heme and form a complex that is not imported into mitochondria, thus providing a form of feedback inhibition (Lathrop and Timko, 1993; Munakata et al., 2004). Following import the signal sequences are proteolytically removed to produce mature enzymes with intrinsically disordered N-termini (Stojanovski et al., 2016; Nomura et al., 2021). This N-terminal extension contains a third conserved HRM that feedback inhibits ALAS1 by binding heme to form a complex targeting ALAS1 for proteolysis by the matrix peptidase chaperone subunit ClpX (Nomura et al., 2021). ClpX also controls ALAS2 turnover (Rondelli et al., 2021), and since HRM 3 is conserved in ALAS2, it seems likely that HRM 3 also mediates ClpX degradation of ALAS2 in a heme-dependent fashion, although this remains to be conclusively demonstrated.

**FIGURE 2:**
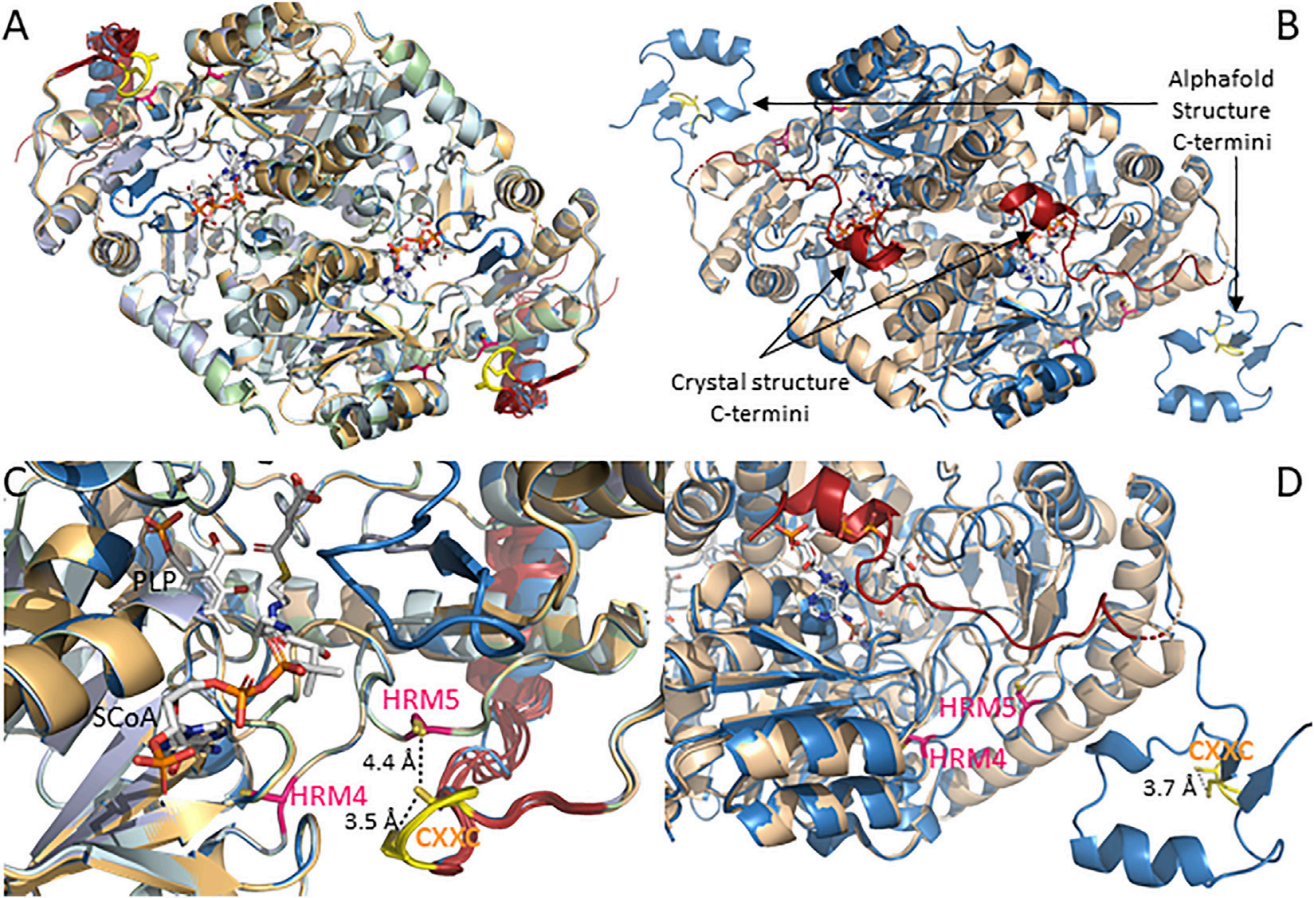
AlphaFold structures for mammalian ALAS1 and ALAS2 reveal C-terminal divergences from the human ALAS2 crystal structure. **(A)**. Alignment of AlphaFold-predicted structures of ALAS1 from human (UniProt accession # P13196), orangutan (UniProt accession Q5R9R9), bovine (UniProt accession A6QLI6), beluga whale (UniProt accession Q9XS79), mouse (UniProt accession Q8VC19), and rat (UniProt accession # P13195). **(B)**. Alignment of human ALAS2 crystal and AlphaFold-predicted structures**.** AlphaFold-predicted structure (UniProt accession # P22557; blue) and crystal structure (PDB code 6HRH; beige with red C-termini). **(C)**. Zoom of panel **(A)**. **(D)**. Zoom of panel **(B)**.

The catalytic core of mammalian ALAS, which is approximately 44 kD in size, contains two additional conserved HRMs, which we designate HRMs 4 and 5. To the best of our knowledge, no studies have yet examined their potential biochemical significance. Along with the human ALAS2 crystal structure, the mammalian ALAS1 and ALAS2 AlphaFold-predicted structures reveal that even though HRMs 4 and 5 are ∼132 amino acids apart in the primary sequence, in the three-dimensional structures the cysteine α-carbons are only 11 Å apart, and most importantly, they are near or at the enzyme surface in proximity to both the active site loop and the extended C-terminus, in conspicuous positions for heme-mediated feedback regulation of the mature enzyme (Figure 2). The positions of HRMs 4 and 5 in the AlphaFold-predicted ALAS structures are virtually indistinguishable from those in the human ALAS2 crystal structure (Figure 2B).

There are, however, important differences in the relative positions of the extended C-termini of the ALAS1 and ALAS2 isozymes as it relates to HRMs 4 and 5. As seen in Figures 2A,C, all six of the currently available AlphaFold-predicted structures for mammalian ALAS1 position the extended C-terminus such that the CXXC motif forms a hairpin loop that brings the cysteine sulfur atoms within ∼ 3.5 Å of each other, suggesting disulfide bond formation and a possible redox sensing role. Furthermore, the CXXC loop is positioned almost directly over HRM5.

In contrast to the consensus positioning of the ALAS1 extended C-terminus over HRMs 4 and 5, the AlphaFold-predicted mammalian ALAS2 structures have more conformational heterogeneity about the C-terminal extension (Figures 2B,D). Moreover, none of the ALAS2 C-terminal extensions align with the ALAS1 C-terminus. Instead, the ALAS2 C-terminal extensions fall into one of three different conformations. In the AlphaFold-predicted structures for orangutan, bovine, beluga whale, and rat ALAS2s, the extended C-terminus folds over the active site to form an “autoinhibited” structure, in excellent alignment with the recently solved human ALAS2 crystal structure (Bailey et al., 2020), but the AlphaFold-predicted human ALAS2 structure places the extended C-terminus away from the catalytic core in what would presumably correspond to an active enzyme conformation. Meanwhile, in the mouse ALAS2, the extended C-terminus adopts a conformation between these two extremes. In all cases the cysteines of the ALAS2 CXXC motifs, like those of the ALAS1 CXXC motifs, are in sufficient proximity to reversibly form disulfides, and thus potentially act as redox sensors. But unlike ALAS1, HRMs 4 and 5 of ALAS2 are not occluded by the C-terminal extension and are thus more available to bind heme in what would presumably be a feedback-inhibited complex.

## A Case for Differential Regulation by the C-Terminal Extensions

Remarkably, in ten out of twelve different mammalian ALAS mitochondrial import presequences AlphaFold predicts the side chains of the cysteines in HRMs 1 and 2 to be almost ideally positioned to act as axial ligands for heme (Supplementary Figure S4). This agrees with experimental evidence demonstrating HRMs 1 and 2 bind heme to feedback inhibit mitochondrial import (Lathrop and Timko, 1993; Goodfellow et al., 2001; Munakata et al., 2004). Further, it leads us to suggest that the predicted conformational differences in the extended C-termini might in turn be experimentally revealed to be accurate predictors of important structural/functional divergences between the two ALAS isozymes.

The AlphaFold structural database currently has nearly a million protein structures available, including complete proteomes for *Homo sapiens* and 47 other species (Jumper et al., 2021; Tunyasuvunakool et al., 2021). These structures are rapidly facilitating an unprecedented understanding of structural biology (Hegedus et al., 2022; Porta-Pardo et al., 2022; Varadi et al., 2022; Wehrspan et al., 2022). Yet, the accuracy of AlphaFold in terms of predicting otherwise unsolved structures is relatively untested since it only became publicly available less than a year ago. AlphaFold is reported to accurately predict not just the highly organized structures observed in crystallized proteins, but also the extent of conformational dynamics or even intrinsic disorder in individual residues or peptides by calculating a per residue confidence score referred to as a predicted local distance difference test (pLDDT) (Tunyasuvunakool et al., 2021). The current interpretation of this score is that it predicts the extent to which a residue is unstructured, meaning a low score should be seen not so much as an indication the structure is inaccurate, but more as an accurate indication of greater conformational dynamics. Because of this AlphaFold should provide important insight into dynamic regulatory structures that have been difficult to crystallize.

The ALAS1/2 conserved CXXC motif is of particular interest since similar motifs act as allosteric redox switches *via* reversible formation of a disulfide bond in many enzymes, including the PLP-dependent enzymes cystathionine β-synthase and human mitochondrial branched chain aminotransferase (Conway et al., 2004; Wouters et al., 2010; Niu et al., 2018; Herbert et al., 2020). The CXXC motif-containing region was only partially resolved in the human ALAS2 crystal structure, implying a high degree of conformational mobility. The AlphaFold pLDDT scores for the six mammalian ALAS2 (and six ALAS1) structures in the public database agree, as they drop from very high confidence to low or even very low for the corresponding amino acids in all species except human ALAS2 (Supplementary Figure S5), in which the extended C-terminus adopts what is presumably an activated enzyme conformation. In this “activated” ALAS2 structure the scores for the CXXC motif are mostly confident, indicating greater structural organization, and with the cysteine side chain sulfur atoms within 3.7 Å of each other, disulfide bond formation is possible. Given all these considerations, if the CXXC motif in the extended C-terminus of ALAS2 acts as a redox switch we would predict that the “activated” structure would be oxidized to the disulfide, while the more disordered autoinhibited structure would be reduced.

The positioning of the human ALAS2 extended C-terminus over the active site leads us to raise the questions as to what the active conformation might look like and how the interconversion between the inhibited and activated conformations might be triggered. The corresponding AlphaFold structure appears to provide a plausible answer to the first of these two questions, but only hints at the answer to the second. Binding of the β-subunit of succinyl-CoA synthetase (Furuyama and Sassa, 2000; Bishop et al., 2012; Bishop et al., 2013) and/or other heme biosynthetic enzymes might promote activation (Medlock et al., 2015). A novel, but certainly not mutually exclusive, possibility supported by the structures analyzed here is that the CXXC motif acts as a redox sensor to modulate conformational dynamics about the extended C-terminus.

In contrast to ALAS2, a crystal structure for ALAS1 has not yet been reported, and the AlphaFold-predicted structures indicate only one conformation for the ALAS1 extended C-terminus. Yet, the CXXC motif is conserved in ALAS1, and if it has a redox switching function then some degree of conformational perturbation presumably occurs to form an autoinhibited conformation or to alter the dynamics about the active site loop, which controls the catalytic rate. This latter possibility is attractive as it would be consistent with the anti-correlation between the active site loop and C-terminal extension of ALAS2 during molecular dynamics simulations (Na et al., 2018). Additionally, the shielding of the otherwise solvent exposed HRMs 4 and 5 by the ALAS1 C-terminal extension suggests an alternative conformation that would allow heme access to feedback inhibit the enzyme. Given these considerations we posit that the ALAS1 structures represent an activated form wherein the CXXC motif is oxidized to the disulfide and positioned to prevent allosteric feedback inhibition by heme. Reduction of the CXXC motif would then facilitate a conformation change allowing heme to allosterically feedback inhibit ALAS1 *via* HRMs 4 and/or 5. A more prominent role of redox sensing in ALAS1 is in part attractive due to the role of ALAS1 in producing heme specifically for hemoproteins catalyzing redox chemistry, such as cytochrome P450 enzymes, catalase, and superoxide dismutase.

## Conclusion and Outlook

In summary, based upon the alignment of the ALAS1 structures we put forth the following postulates: 1) HRMs 4 and/or 5 facilitate feedback inhibition of ALAS1; 2) under oxidizing conditions, the CXXC motif forms a disulfide bond that causes the C-terminal extension to fold over HRMs 4 and 5 such that it sterically prevents hemin binding and feedback inhibition; 3) under non-oxidizing conditions, the CXXC motif is reduced and adopts an alternative conformation wherein HRMs 4 and 5 are exposed to provide feedback inhibition by excess heme. Stated more concisely, feedback inhibition of ALAS1 by heme is dependent upon cellular redox status.

Based on the alignment of the ALAS2 structures we put forth the following postulates: 1) the C-terminal extension of ALAS2 adopts two different conformations, neither of which prevents feedback regulation via heme binding to HRMs 4 and 5. 2) In ALAS2 oxidizing conditions cause disulfide bond formation in the CXXC motif and movement of the extended C-terminus not over HRMs 4&5 but instead to a more equatorial and activated position relative to the enzyme, thereby relieving the autoinhibition observed when the extended C-terminus folds over the active site. Stated more succinctly, heme and redox status independently regulate ALAS2 activity.

These postulates are not incompatible with the possibility of protein-protein interactions regulating activity. Of course, experimental data will be required to further support or refine the views presented here, but whatever the outcome the remarkably divergent structures discussed here will likely represent a key test of the capacity of AlphaFold to discern fine structural differences and facilitate prediction of allostery in all enzymes, including those dependent upon PLP for functionality.
