# Supplementary material for: An Extended C-Terminus, the Possible Culprit for Differential Regulation of 5-Aminolevulinate Synthase Isoforms
Source: Front Mol Biosci. 2022 Jul 14;9:920668. doi: 10.3389/fmolb.2022.920668 (PMC9329541; doi:10.3389/fmolb.2022.920668)
Supplement: Supplementary file 1 [file DataSheet1.pdf]

Supplementary Material

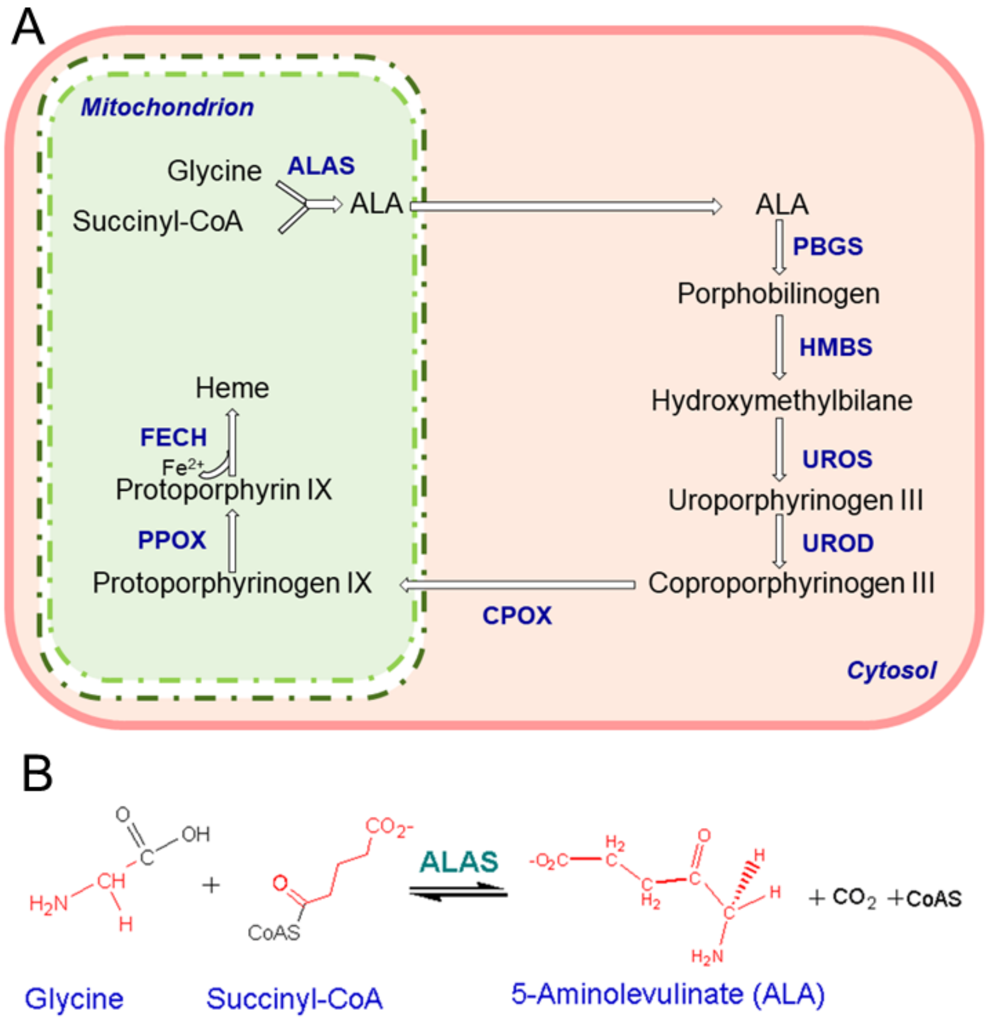

Supplementary Figure 1. Enzymatic steps of the heme biosynthetic pathway in metazoa (Panel A) and ALAS-catalyzed reaction (Panel B). The abbreviations of the names for the eight enzymes are indicated in blue font, and the substrates/products are in black font. Abbreviations: ALA, 5-aminolevulinate; ALAS, ALA synthase; CPOX, coproporphyrinogen oxidase; FECH, ferrochelatase; HMBS, hydroxymethylbilane synthase; PBGS, porphobilinogen synthase; PPOX, protoporphyrinogen oxidase; UROD, uroporphyrinogen III decarboxylase; UROS, uroporphyrinogen III synthase.

```
CLUSTAL O(1.2.4) multiple sequence alignment


ALAS1 Monodelphis domestica       ------------------------------------------------------------ 0
ALAS1 Sarcophilus harrisii        ------------------------------------------------------------ 0
ALAS1 Phascolarctos cinereus      ------------------------------------------------------------ 0
ALAS1 Vombatus ursinus            ------------------------------------------------------------ 0
ALAS1 Bos taurus                  MRSCPSECILGRQGSRPEGSCRQGQGLVRRFHLGTCGLRH---------------LRRVP 45
ALAS1 Meriones unguiculatus       ------------------------------------------------------------ 0
ALAS1 Rattus norvegicus           ------------------------------------------------------------ 0
ALAS1 Mus musculus                ------------------------------------------------------------ 0
ALAS1 Ochotona curzoniae          MASRNVRLCVPGSEIHSKFHKSRNQLEERN--WHSDLERP-------------------K 39
ALAS1 Oryctolagus cuniculus       MRTAPRRCILGGSIRGLRLLADKGKRSC-G--HLGL-----------SARRRGRRLAECP 46
ALAS1 Cavia porcellus             ------------------------------------------------------------ 0
ALAS1 Chinchilla lanigera         ------------------------------------------------------------ 0
ALAS1 Nannospalax galili          ------------------------------------------------------------ 0
ALAS1 Castor canadensis           ------------------------------------------------------------ 0
ALAS1 Delphinapterus leucas       ---------------------------------------------MTVASSLASLLHLGG 15
ALAS1 Homo sapiens                ------------------------------------------------------------ 0
ALAS1 Pan troglodytes             ------------------------------------------------------------ 0
ALAS1 Macaca mulatta              ------------------------------------------------------------ 0
ALAS1 Equus caballus              ------------------------------------------------------------ 0
ALAS1 Odobenus rosmarus           MRSAPSGCILGRRRSRPEASRRQGHRVDSS--WFGCLPQPPPPPPPPPPPPPPPPPRQSS 58
ALAS1 Lontra canadensis           ------------------------------------------------------------ 0
ALAS1 Hyaena hyaena               ------------------------------------------------------------ 0
ALAS1 Acinonyx jubatus            ------------------------------------------------------------ 0
ALAS1 Panthera tigris             ------------------------------------------------------------ 0
ALAS1 Panthera pardus             ------------------------------------------------------------ 0
ALAS1 Lynx canadensis             ------------------------------------------------------------ 0
ALAS1 Puma yagouaroundi           ------------------------------------------------------------ 0
ALAS1 Puma concolor               ------------------------------------------------------------ 0
ALAS1 Felis catus                 ------------------------------------------------------------ 0
ALAS1 Ailuropoda melanoleuca      ------------------------------------------------------------ 0
ALAS1 Canis lupus dingo           MRSAPSGSILGRLRSRPEASRRQGQRVGVI--WLGCPPPPPPP--------PPLPPPQSP 50
ALAS2 Cavia porcellus             ------------------------------------------------------------ 0
ALAS2 Chinchilla lanigera         -------------MPGPNPQHPS--VF-------WFVL-----SA-------------GQ 20
ALAS2 Rattus norvegicus           ------------------------------------------------------------ 0
ALAS2 Mus musculus                ------------------------------------------------------------ 0
ALAS2 Nannospalax galili          ------------------------------------------------------------ 0
ALAS2 Panthera tigris             MISTSLGRAACTGWEGSNTGMTL--GFWPGLHDGLSSL-----AH-------------LL 40
ALAS2 Panthera pardus             ------------------------------------------------------------ 0
ALAS2 Puma concolor               ------------------------------------------------------------ 0
ALAS2 Lynx canadensis             ------------------------------------------------------------ 0
ALAS2 Puma yagouaroundi           ------------------------------------------------------------ 0
ALAS2 Acinonyx jubatus            ------------------------------------------------------------ 0
ALAS2 Felis catus                 ------------------------------------------------------------ 0
ALAS2 Lontra canadensis           ------------------------------------------------------------ 0
ALAS2 Canis lupus dingo           ------------------------------------------------------------ 0
ALAS2 Ailuropoda melanoleuca      ------------------------------------------------------------ 0
ALAS2 Odobenus rosmarus           ------------------------------------------------------------ 0
ALAS2 Castor canadensis           ------------------------------------------------------------ 0
ALAS2 Ochotona curzoniae          ------------------------------------------------------------ 0
ALAS2 Oryctolagus cuniculus       ------------------------------------------------------------ 0
ALAS2 Homo sapiens                ------------------------------------------------------------ 0
ALAS2 Pan troglodytes             ------------------------------------------------------------ 0
ALAS2 Equus caballus              ------------------------------------------------------------ 0
ALAS2 Bos taurus                  ------------------------------------------------------------ 0
ALAS2 Delphinapterus leucas       -----------MLWDGT---RGR--GCFPGLHGGISSL-----PH-------------LM 26
ALAS2 Sarcophilus harrisii        MQRHEGGGARELRTAPAQDFSLI--CPRAGGRGQGQKYRLWCRTS-------------RS 45
ALAS2 Phascolarctos cinereus      ------------------------------------------------------------ 0
ALAS2 Vombatus ursinus            ------------------------------------------------------------ 0
```

```
ALAS1 Monodelphis domestica       ---------METIVRRCPFLSRVPQTFLQKAGK--SLLFYAQNCPKMMEVVSKPASRAMA 49
ALAS1 Sarcophilus harrisii        ---------METIVRRCPFLSRVPQTFLQKAGK--SLLFYAQNCPKMMEIGSKPASRTVT 49
ALAS1 Phascolarctos cinereus      ---------METIVRRCPFLSRVPQTFLQKAGK--SLLFYAQNCPKMMEIASKPASRAMT 49
ALAS1 Vombatus ursinus            ---------METIVRRCPFLSRVPQTFLQKAGK--SLLFYAQNCPKMMEIGSKPASRAMT 49
ALAS1 Bos taurus                  RAGSVYLREMETVVRRCPFLSRVPQAFLQKAGK--SLLFYAQNCPKMMEIGAKPAPRALS 103
ALAS1 Meriones unguiculatus       ---------METVVRRCPFLSRVPQAFLQKAGK--SLLFYAQNCPKMMEVGAKPAPRTMS 49
ALAS1 Rattus norvegicus           ---------METVVRRCPFLSRVPQAFLQKAGK--SLLFYAQNCPKMMEVGAKPAPRTVS 49
ALAS1 Mus musculus                ---------METVVRRCPFLSRVPQAFLQKAGK--SLLFYAQNCPKMMEVGAKPAPRTLS 49
ALAS1 Ochotona curzoniae          TRSQHTSWTMETVVRRCPFLSRVPQAFLQKAGK--SLLFYAQNCPKMMEFGAKPAPRALS 97
ALAS1 Oryctolagus cuniculus       AQELAHSLDMETVVRRCPFLSRVPQAFLQKAGK--SLLFYAQNCPKMMEAGAKPAPRALS 104
ALAS1 Cavia porcellus             ---------METVVRRCPFLSRVPQAFLQKAGK--SLLFYAQNCPKMMEVGAKPAPRGLS 49
ALAS1 Chinchilla lanigera         ---------METVVRRCPFLSRVSQAFLQKAGK--SLLFYAQNCPKMMEVGAKPAPRGLS 49
ALAS1 Nannospalax galili          ---------METVVRRCPFLSRVPQAFLQKAGK--SLLFYAQNCPKMMEVGVKPGPRALS 49
ALAS1 Castor canadensis           ---------METVVRRCPFLSRVSQAFLQKAGK--SLLFYAQNCPKMMEVGAKPAPRALS 49
ALAS1 Delphinapterus leucas       CQGSVHLRDMETVVRRCPFLSRVPQAFLQKAGK--SLLFYAQNCPKMMEVGAKPAPRALS 73
ALAS1 Homo sapiens                ---------MESVVRRCPFLSRVPQAFLQKAGK--SLLFYAQNCPKMMEVGAKPAPRALS 49
ALAS1 Pan troglodytes             ---------MESVVRRCPFLSRVPQAFLQKAGK--SLLFYAQNCPKMMEVGAKPAPRALS 49
ALAS1 Macaca mulatta              ---------METVVRRCPFLSRVPQAFLQKAGK--SLLFYAQNCPKMMEVGAKPAPRALS 49
ALAS1 Equus caballus              ---------METVVRRCPFLSRVPQAFLQKAGK--SLFFYAQNCPKMMEVGAKPAPRAVS 49
ALAS1 Odobenus rosmarus           AQDQCTLSDMETVVRRCPFLSRVPQAFLQKAGK--SLLFYAQNCPKMMEVGAKPAPRALS 116
ALAS1 Lontra canadensis           ---------METVVRRCPFLSRVPQAFLQKAGK--SLLFYAQNCPKMMEFGAKPATRALS 49
ALAS1 Hyaena hyaena               ---------METVVRRCPFLSRVPQAFLQKAGK--SLLFYAQNCPKMMEFGAKPAARALS 49
ALAS1 Acinonyx jubatus            ---------METVVRRCPFLSRVPQAFLQKAGK--SLLFYAQNCPKMMEVGAKPAPRALS 49
ALAS1 Panthera tigris             ---------METVVRRCPFLSRVPQAFLQKAGK--SLLFYAQNCPKMMEVGAKPAPRALS 49
ALAS1 Panthera pardus             ---------METVVRRCPFLSRVPQAFLQKAGK--SLLFYAQNCPKMMEVGAKPAPRALS 49
ALAS1 Lynx canadensis             ---------METVVRRCPFLSRVPQAFLQKAGK--SLLFYAQNCPKMMEVGAKPAPRALS 49
ALAS1 Puma yagouaroundi           ---------METVVRRCPFLSRVPQAFLQKAGK--SLLFYAQNCPKMMEVGAKPAPRALS 49
ALAS1 Puma concolor               ---------METVVRRCPFLSRVPQAFLQKAGK--SLLFYAQNCPKMMEVGAKPAPRALS 49
ALAS1 Felis catus                 ---------METVVRRCPFLSRVPQAFLQKAGK--SLLFYAQNCPKMMEVGAKPAPRALS 49
ALAS1 Ailuropoda melanoleuca      ---------METVVRRCPFLSRVPQAFLQKAGK--SLLFYAQNCPKMMEVGAKPAPRALS 49
ALAS1 Canis lupus dingo           AQDQCTLSDMETVVRRCPFLSRVPQAFLQKAGK--SLLFYAQNCPKMMEIGAKPAPRALS 108
ALAS2 Cavia porcellus             ------MVAIATLLHYCPVLARTPTGLLGKVIKIHQFLFGTRRCPILATQGSTCSQIYLK 54
ALAS2 Chinchilla lanigera         QEFGLKMVAVAMLLQYCPVLARTPTGLLGKVIKTHQFLLGTGRCPILATQGPTCSQIHFK 80
ALAS2 Rattus norvegicus           ------MVAAAMLLRSCPVLSKGPTGLLGKVAKTYQFLFGIGRCPILATQGPTCSQIHLK 54
ALAS2 Mus musculus                ------MVAAAMLLRSCPVLSQGPTGLLGKVAKTYQFLFSIGRCPILATQGPTCSQIHLK 54
ALAS2 Nannospalax galili          ------MVTAAVLLQSCPVLSRGPTGLLGKVAKTYQFLFGTGRCPILATQGPTYSQIHLK 54
ALAS2 Panthera tigris             QDFGLSMVAAVMLLQCCPVFSRGHIGLLGKMIKTHQFLFGIGRCPILTTQGPTSSQNHLK 100
ALAS2 Panthera pardus             ------MVAAVMLLQCCPVFSRGHIGLLGKMIKTHQFLFGIGRCPILTTQGPTSSQNHLK 54
ALAS2 Puma concolor               ------MVAAVMLLQCCPVFSRGHIGLLGKMIKTHQFLFGIGRCPILTTQGPTSSQIHLK 54
ALAS2 Lynx canadensis             ------MVAAVMLLQCCPVFSRGHIGLLGKMIKTHQFLFGIGRCPILTTQGPTSSQIHLK 54
ALAS2 Puma yagouaroundi           ------MVAAVMLLQCCPVFSRGHIGLLGKMIKTHQFLFGIGRCPILTTQGPTSSQIHLK 54
ALAS2 Acinonyx jubatus            ------MVAAVMLLQCCPVFSRGHIGLLGKMIKTHQFLFGIGRCPILTTQGPTSSQIHLK 54
ALAS2 Felis catus                 ------MVAAVMLLQCCPVFSRGHIGLLGKMIKTHQFLFGIGRCPILTTQGPTSSQIHLK 54
ALAS2 Lontra canadensis           ------MVAAALLLQCCPVLARGHTGPLGKMLKSHQFLFGIGRCPILATQGPTCSQIHLK 54
ALAS2 Canis lupus dingo           ------MVVVAMLLQCCPVLARDHIGLLGKIIKTHQFLFGIGRCPILATQRPTFSQIHLK 54
ALAS2 Ailuropoda melanoleuca      ------MVAAAMLLQCCPVLARGHIGLLGKMIKTHQVLFGIGRCPILATQGPNCSQIHLK 54
ALAS2 Odobenus rosmarus           ------MVAAAMLLQCCPVLARGHIGLLGKMIKTHQFLFGPGRCPILATYGPNCSQIHLK 54
ALAS2 Castor canadensis           ------MVAAAVLLQCCPVLARGPTGLLGKVVKIHPFLFGIGHCPILATQGPTCSQIHLK 54
ALAS2 Ochotona curzoniae          ------MVAAAVLLRCCPVLAQGPTGFLRKVTKTHQFLFGTGRCPILATQGPTCSQIHLK 54
ALAS2 Oryctolagus cuniculus       ------MVAAAMLLRCCPVLAQSPTGLLRKVTKAHQFLFGIGRCPILAAQGPACSQIHLK 54
ALAS2 Homo sapiens                ------MVTAAMLLQCCPVLARGPTSLLGKVVKTHQFLFGIGRCPILATQGPNCSQIHLK 54
ALAS2 Pan troglodytes             ------MVTAAMLLQCCPVLARGPTSLLGKVVKTHQFLFGIGRCPILATQGPNCSQIHLK 54
ALAS2 Equus caballus              ------MVAAAILLQRCPVLARGPTGILGKVIKTHQFLFGIGRCPILATHEPICSQIHLK 54
ALAS2 Bos taurus                  ------MVTAAMLLQRCPVLIRSPTGLLGKMIKTHQFLFGIGRCPILATQGPSFSQIHLK 54
ALAS2 Delphinapterus leucas       QNFGLKRVAAAMLLQRCPVLIRSPTAILGKMIKTHQFLIGIGRCPILATQGTTCSQIHLK 86
ALAS2 Sarcophilus harrisii        SSVQGNRPSMASFLQRCPALAWDRVAFLKKIR--PQLLLNAHRCPVMVAQGLVTSSPEGK 103
ALAS2 Phascolarctos cinereus      ---------MASFLQRCPAMAWDRATLLKKTR--PQLLFSAQRCPVMVARGLATSSPESK 49
ALAS2 Vombatus ursinus            ---------MASFLQRCPAMARDRATFLKKTR--PQLLLSAQRCPVMVARGLATSSPGSK 49
                                               .:: ** :        * *       .::    .** :          .
                    ALAS1 mature enzyme →                                    ALAS2 mature enzyme
ALAS1 Monodelphis domestica       TSPTNFQQVKETPPANEKDKTSKAVVQPAGDSQ--------QATGGSQLPSGHPSSATSQ 101
ALAS1 Sarcophilus harrisii        TSPSHFQQVKETPPTNEKDKTSKTVVQHADGSQ--------QTTGGSQLPSGHPSSAASQ 101
ALAS1 Phascolarctos cinereus      TSPTHFQQVEETPPVNEKNKTSKTVVQHADSSQ--------QTAGGSQLPSGHPASATNQ 101
ALAS1 Vombatus ursinus            TSPTHFQQVKETPPANEKDKTSKTVVQDADDSQ--------QTAGGSQLPSGHPTSATSQ 101
ALAS1 Bos taurus                  TSAVLCQQVTETPPANEKDKAAKAEVQQAPDGSQQAPDGSQQTADGTQLPSGHPSLASSQ 163
ALAS1 Meriones unguiculatus       TSAVHCQQIKETPPANEKEKTAKATAHQAPDESQMA-----QNPDGTQLPAGHPSPAASQ 104
ALAS1 Rattus norvegicus           TSAAQCQQVKETPPANEKEKTAKAAVQQAPDESQMA-----QTPDGTQLPPGHPSPSTSQ 104
ALAS1 Mus musculus                TSAVHCQQVKETPPANEKEKTAKAAVQQAPDESQMA-----QTPDGTQLPSGHPSPATSQ 104
ALAS1 Ochotona curzoniae          TSAGHCQQIKETPPASEKDKTAQAEVQQAPDGTQ-------RPLDGTQLPAGHPSPVPSQ 150
```

```
ALAS1 Oryctolagus cuniculus   TSAGHCQQIKETPPASEKDKTAQAEVQQAPASSQ-------RPADGTQLPSGHPSPASSQ 157
ALAS1 Cavia porcellus         TSAVHCQQIKETPPASEKDKTAQAKVQHTPDGSQ-------QTPDGTQLPPGHPSPATSQ 102
ALAS1 Chinchilla lanigera     TSAVPCQQVKETPPASGKDKTAKAKVQQAPDRSQ-------ETPDGTQPPPGHPSPATSQ 102
ALAS1 Nannospalax galili     TSAVPCQQIKETPPANEKEKTAKATVQQAPDGSQ-------QTPDGTQLLSGHPLPATSQ 102
ALAS1 Castor canadensis       TSAVHCQQIKETPPANEKDKTAKAKIQQPPDRSQ-------QTPDGTQLLSGHPSPATSQ 102
ALAS1 Delphinapterus leucas   ASAVLCQQVKETPPANEKDKTAKAKVQQAPDGSQ-------QTPDGTQLPSGHPSLAASQ 126
ALAS1 Homo sapiens            TAAVHYQQIKETPPASEKDKTAKAKVQQTPDGSQ-------QSPDGTQLPSGHPLPATSQ 102
ALAS1 Pan troglodytes         TAAVHYQQIKETPPASEKDKTAKAKVQQTPDGSQ-------QSPDGTQLPSGHPLPATSQ 102
ALAS1 Macaca mulatta          TAAVHYQQIKETSPASEKDKTAKAKVQQAPDGSQ-------QSPDGTQLPSGHPLPATSQ 102
ALAS1 Equus caballus          TSSLCCQQVTETPPASEKDKTAKAETQQAPDGSQ-------QTPDGTQLPSEHPSLTTSQ 102
ALAS1 Odobenus rosmarus       TSTVPCQQVKETPPANEKDKTAKAEVEQAADGSQ-------QTPDGTQLSSGHPSLATSQ 169
ALAS1 Lontra canadensis       TSAVHCQQVKETPPANEKDKTAKAEVQQAADGSQ-------QTPHGTQCPSGHPSLATSQ 102
ALAS1 Hyaena hyaena           TSAVHCQQVKETPPANEKDKTAKAEGQQVPDGSQ-------KTPDGTQLPSGHPSLATSQ 102
ALAS1 Acinonyx jubatus        TSTVHCQQVKETPPANEKDKTAKAEGQQAPDGSQ-------QTPDGTQIPSGHPSLATSQ 102
ALAS1 Panthera tigris         TSTVHCQQVKETPPANEKDKTAKAEGQQVPDGSQ-------QTPDGTQIPSGHPSLATSQ 102
ALAS1 Panthera pardus         TSTVHCQQVKETPPANEKDKTAKAEGQQVPDGSQ-------QTPDGTQIPSGHPSLATSQ 102
ALAS1 Lynx canadensis         TSTVHCQQVKETPPANEKDKTAKAEGQQAPDGSQ-------QTPDGTQIPSGHPSLATSQ 102
ALAS1 Puma yagouaroundi       TSTVHCQQVKETPPANEKDKTAKAEGQQAPDGSQ-------QTPDGTQIPSGHPSLATSQ 102
ALAS1 Puma concolor           TSTVHCQQVKETPPANEKDKTAKAEGQQAPDGSQ-------QTPDGTQIPSGHPSLATSQ 102
ALAS1 Felis catus             TSTVHCQQVKETPPANEKDKTAKAEGQQAPDGSQ-------QTPDGTQIPSGHPSLATSQ 102
ALAS1 Ailuropoda melanoleuca  TSAVHCQQVKETPPANEKDKTAKAEVQQAADGSQ-------QTPDGTQLPSGHPSLATSQ 102
ALAS1 Canis lupus dingo       TSAVHCQQVKETPPANEKDKTAKAEVQQTADGSQ-------QTPDGTQLPSGHSSLATSQ 161
ALAS2 Cavia porcellus         -------------------------------------------------A-TKAGWGSPS 64
ALAS2 Chinchilla lanigera     -------------------------------------------------A-TKAGEDSPS 90
ALAS2 Rattus norvegicus       -------------------------------------------------A-TKAGADSPS 64
ALAS2 Mus musculus            -------------------------------------------------A-TKAGGDSPS 64
ALAS2 Nannospalax galili      -------------------------------------------------A-TKAGGDSSS 64
ALAS2 Panthera tigris         -------------------------------------------------A-TKAGGDSPS 110
ALAS2 Panthera pardus         -------------------------------------------------A-TKAGGDSPS 64
ALAS2 Puma concolor           -------------------------------------------------A-TKAGGDSPS 64
ALAS2 Lynx canadensis         -------------------------------------------------A-TKAGGDSPS 64
ALAS2 Puma yagouaroundi       -------------------------------------------------A-TKAGGDSPS 64
ALAS2 Acinonyx jubatus        -------------------------------------------------A-TKAGGDSPS 64
ALAS2 Felis catus             -------------------------------------------------A-TKAGGDSPS 64
ALAS2 Lontra canadensis       -------------------------------------------------A-TKAGGDSPS 64
ALAS2 Canis lupus dingo       -------------------------------------------------A-TKAGGDSPP 64
ALAS2 Ailuropoda melanoleuca  -------------------------------------------------A-TKAGGDSQS 64
ALAS2 Odobenus rosmarus       -------------------------------------------------A-TKAGGDSPS 64
ALAS2 Castor canadensis       -------------------------------------------------A-TKAGGDSSS 64
ALAS2 Ochotona curzoniae      -------------------------------------------------E-TKAGENTPP 64
ALAS2 Oryctolagus cuniculus   -------------------------------------------------A-TKAGENSSS 64
ALAS2 Homo sapiens            -------------------------------------------------A-TKAGGDSPS 64
ALAS2 Pan troglodytes         -------------------------------------------------A-TKAGGDSPS 64
ALAS2 Equus caballus          -------------------------------------------------A-TKAGGDSPS 64
ALAS2 Bos taurus              -------------------------------------------------A-TKAGGDSPS 64
ALAS2 Delphinapterus leucas   -------------------------------------------------A-TKAGGDSPS 96
ALAS2 Sarcophilus harrisii    -------------------------------------------------ETKAQASGSLS 114
ALAS2 Phascolarctos cinereus  -------------------------------------------------EAEVQAYGSPS 60
ALAS2 Vombatus ursinus        -------------------------------------------------EAEVQACGSPS 60

ALAS1 Monodelphis domestica   GTASKCPFLAAQLNQGTSNVFRKASLELQEDVQEMQAVRKEVAQTPINPTVINLKTDGED 161
ALAS1 Sarcophilus harrisii    GTASKCPFLAAQMNQGTSNVFRKASLELQEDVQEMQAVRKEVAQTPINPTVINLKTGRED 161
ALAS1 Phascolarctos cinereus  GTASKCPFLAAQMNQGTSNVFRKASLELQEDVQEMQAVRKEVAQTPINPTVINLKAGRED 161
ALAS1 Vombatus ursinus        GTASKCPFLAAQMNQGTSNVFRKASLELQEDVQEIQAVRKEVAQPPVNPAVINLQTGRED 161
ALAS1 Bos taurus              GTGSKCPFLAAEMSQGGSSVFRKASLALQEDVQEMHAVREEVAQTSVNPSVINVKTEGGE 223
ALAS1 Meriones unguiculatus   GSGSKCPFLAAQLSQTGSSVFRKASLELQEDVQEMHAVRKEVAQSPVTPSVITVKTDRED 164
ALAS1 Rattus norvegicus       SSGSKCPFLAAQLSQTGSSVFRKASLELQEDVQEMHAVRKEVAQSPVLPSLVNAKRDGEG 164
ALAS1 Mus musculus            GSGSKCPFLAAQLSQTGSSVFRKASLELQEDVQEMHAVRKEAAQSPVPPSLVNVKTDGED 164
ALAS1 Ochotona curzoniae      GTASKCPFLAAQMSQTGSSVFRKASLELQEDVQEMHAVRKEVAESSGNPSVISVKTDEGD 210
ALAS1 Oryctolagus cuniculus   GAGSKCPFLAAQMSQGGSSVVRKASLELQEDVQEIHAVRKEAAQSSVNASVISVKTDGED 217
ALAS1 Cavia porcellus         GSASKCPFLAAQMSQKGSSVFCKASLELQEDVQEMHAVRKEVAQTSAKPSVITVKTDGEE 162
ALAS1 Chinchilla lanigera     GTASRCPFLAAQMSQKGSSVFCKASLELQEDVQEMHAVRKEVAQTSVNPSVINMKTDGED 162
ALAS1 Nannospalax galili      GSGSKCPFLAAQMSQTGSSVFRKASLELQEDVQEMHAVRKEVAKTSVTPSVINVKTDEED 162
ALAS1 Castor canadensis       GSASKCPFLAAQMNQMGSNVFRKASLELQEDVQEMHAVRKEVAQTSVNPSVINVKTDGED 162
ALAS1 Delphinapterus leucas   GTASKCPFLAAQMSQGGSSVFCKASLELQEDVQEMHAVREEVAQTSVNPSVISVKTDGGE 186
ALAS1 Homo sapiens            GTASKCPFLAAQMNQRGSSVFCKASLELQEDVQEMNAVRKEVAETSAGPSVVSVKTDGGD 162
ALAS1 Pan troglodytes         GTASKCPFLAAQMNQRGSSVFCKASLELQEDVQEMNAVRKEVAETSAGPSVVSVKTDGGD 162
ALAS1 Macaca mulatta          GTASKCPFLAAQMNQRGSSVFCKASLELQEDVQEMNAVRKEVAETSASPSVVSVKTDGGD 162
ALAS1 Equus caballus          GTASKCPFLAAQMSQRGSSVFCKASLELQEDVQEMHAVRKEVAQTSANPSVISVKTDGGE 162
ALAS1 Odobenus rosmarus       GTASKCPFLAAQMSQKGSSVFCKASLELQEDVQEMHAVRKEVAQTSVNPSVISVKADEGE 229
```

```
ALAS1 Lontra canadensis       GTASKCPFLAAQMSQKGSSVFRKASLELQEDVQEMHAVRKEVAQTSVNPSVISVKADEGE 162
ALAS1 Hyaena hyaena           GTASKCPFLAAQMSQKGSSVFRKASLELQEDVQEMHAVRKEVAQTSVNPSVISVKTDGGE 162
ALAS1 Acinonyx jubatus        GTASRCPFLAAQMSQKGSSVFCKASLELQEDVQEMHAVRKEVAQTSVNPSEISVKTDSGE 162
ALAS1 Panthera tigris         GTASKCPFLAAQMSQKGSSVFCKASLELQEDVQEMHAVRKEVAQTSVNPSVISVKTDSGE 162
ALAS1 Panthera pardus         GTASKCPFLAAQMSQKGSSVFCKASLELQEDVQEMHAVRKEVAQTSVNPSVISVKTDSGE 162
ALAS1 Lynx canadensis         GTASRCPFLAAQMSQKGSSVFCKASLELQEDVQEMHAVRKEVAQTSVNPSVISVKTDSGE 162
ALAS1 Puma yagouaroundi       GTASRCPFLAAQMSQKGSSVFCKASLELQEDVQEMHAVRKEVAQTSVNPSVISVKTDSGE 162
ALAS1 Puma concolor           GTASRCPFLAAQMSQKGSSVFCKASLELQEDVQEMHAVRKEVAQTSVNPSVISVKTDSGE 162
ALAS1 Felis catus             GTASKCPFLAAQMSQKGSSVFCKASLELQEDVQEMHAVRKEVAQTSVNPGVISVKTDSGE 162
ALAS1 Ailuropoda melanoleuca  GTASKCPFLAAQMSQKGSNVFRKASLELQEDVQEMHAVRKEVAQTSVNSSVISVKADEGE 162
ALAS1 Canis lupus dingo       GTASKCPFLAAQMSQKGSSVFRKASLELQEDVQEMHAVRKEVAQTSVNPSVISVKADGGD 221
ALAS2 Cavia porcellus         LANGHCPFMLSELQHGRSKIVQKAAPEVQEDVKTFKT------DLPSSLVSSSLK----- 113
ALAS2 Chinchilla lanigera     WAKSHCPFMLSELQDRKSKIVQRAAPEVQEDVKTFKTVPYFLSDLLSFMESTTRS----- 119
ALAS2 Mus musculus            WAKSHCPFMLSELQDRKSKIVQRAAPEVQEDVKTFKT------DLLSTMDSTTRS----- 113
ALAS2 Nannospalax galili      WARSHCPFMLSELQDGNSKIVQKAAPEVQEDVKAFKT------DLLNSLDSSIPR----- 113
ALAS2 Panthera tigris         WAKSHCPFMLSELQDGKSKIVQKAAPEVQEDVKTFKT------DLHSSLASTSLR----- 159
ALAS2 Panthera pardus         WAKSHCPFMLSELQDGKSKIVQKAAPEVQEDVKTFKT------DLHSSLASTSLR----- 113
ALAS2 Puma concolor           WAKNHCPFMLSELQDGKSKIVQKAAPEVQEDVKTFKT------DLHSSLASTSLR----- 113
ALAS2 Lynx canadensis         WAKSHCPFMLSELQDGKSKIVQKAAPEVQEDVKTFKT------DLHSSLASTSLR----- 113
ALAS2 Puma yagouaroundi       WAKSHCPFMLSELQDGKSKIVQKAAPEVQEDVKTFKT------DLHSSLASTSLR----- 113
ALAS2 Acinonyx jubatus        WAKSHCPFMLSELQDGKSKIVQKAAPEVQEDVKTFKT------DLHSSLASTSLR----- 113
ALAS2 Felis catus             WAKSHCPFMLSELQDGKSKIVQKAAPEVQEDVKTFKT------DLHSSLASTSLR----- 113
ALAS2 Lontra canadensis       WAKSHCPFMLSELQDGKSKIVQKAAPEVQEDVKIFKT------DLPRSLASTSLR----- 113
ALAS2 Canis lupus dingo       WAKSPCPFMLSELQDGKSKIVQKAAPEVQEDVKTFKT------DLPRSPASTSLR----- 113
ALAS2 Ailuropoda melanoleuca  WAKSHCPFMLSELQDGKSKIVQKAAPEVQEDVKTFKT------DTPRSLASTSLR----- 113
ALAS2 Odobenus rosmarus       WAKSHCPFMLSELQDGKSKIVQKAAPEVQEDVKTFKT------DLPRSLASTSLR----- 113
ALAS2 Castor canadensis       WAKGHCPFMLSELQDGKSKIVQKAAPEVQEDVKTFKT------DLLSSLASTSPR----- 113
ALAS2 Ochotona curzoniae      WTKGHCPFMLSELQDGKSKIVQKAAPEVQEDVKAFKT------DLPYFLASTSPR----- 113
ALAS2 Oryctolagus cuniculus   WAKGHCPFMLSELQDGKSKIVQKAAPEVQEDVKAFKT------DLPHSLASTSLR----- 113
ALAS2 Homo sapiens            WAKGHCPFMLSELQDGKSKIVQKAAPEVQEDVKAFKT------DLPSSLVSVSLR----- 113
ALAS2 Pan troglodytes         WVKGHCPFMLSELQDGKSKIVQKAAPEVQEDVKAFKT------DLPSSLVSVSLR----- 113
ALAS2 Equus caballus          WAKSHCPFMLSELQDGKSKIVQKAAPEVQEDVKTFKT------DLPSSLTSTSLR----- 113
ALAS2 Bos taurus              WAKSHCPFMLLELQDGKSKIVQKAAPEVQEDVKTFKT------DLPTSLASTSLK----- 113
ALAS2 Delphinapterus leucas   WAKSHCPFMLLELQDGKSKIVQKAAPEVQEDVKTFKT------DLPISLASTSLR----- 145
ALAS2 Sarcophilus harrisii    LAKGQCPFMESELQTGKSNIVLKAVPEILEDVKPFKPVPITC--LVQEMQSSLKK----- 167
ALAS2 Phascolarctos cinereus  LAKGQCPFMESELQAGKSKILQKAGPEIQEDVKTFKAVPISC--LVQEIQSTLRK----- 113
ALAS2 Vombatus ursinus        LAKGQCPFMESDLQAGKSKIMLKAGPEIQEDVKTFKAVPISC--LVQEIQSSLRK----- 113
                                 . ***:  ::.   *.:. :*   : ***: ::

ALAS1 Monodelphis domestica   QNGLLRNFQDLMQKQRPQRVSHLLQDNLPK---------SVSTFQYDRFFEKKIDEKKSD 212
ALAS1 Sarcophilus harrisii    QSGLLKNFQDLMQKQRPERVSHLLQDNLPK---------SVSTFQYDRFFEKKIDEKKSD 212
ALAS1 Phascolarctos cinereus  PNGLLKNFQDLMQKQRPERVSHLLQDNLPK---------SVSTFQYDRFFEKKIDEKKSD 212
ALAS1 Vombatus ursinus        PNGLLKNFQDLMQKQRPERVSHLLQDNLPK---------SVSTFQYDRFFEKKIDEKKSD 212
ALAS1 Bos taurus              LNGLLKNFQDIMRKQRPERVSHLLQDNLPK---------SVCTFQYDRFFEKKIDEKKND 274
ALAS1 Meriones unguiculatus   PSRLLKNFQDIMRQQRPERVSHLLQDNLPK---------SVSTFQYDHFFEKKIDEKKND 215
ALAS1 Rattus norvegicus       PSPLLKNFQDIMRKQRPERVSHLLQDNLPK---------SVSTFQYDHFFEKKIDEKKND 215
ALAS1 Mus musculus            PSRLLKNFQDIMRKQRPERVSHLLQDNLPK---------SVSTFQYDHFFEKKIDEKKND 215
ALAS1 Ochotona curzoniae      PSSLLKNFQDMMRRQRPERVSHLLQDNLPK---------SVSTFQYDRFFEKKIDEKKND 261
ALAS1 Oryctolagus cuniculus   PSGLLKNFQDIMRKQRPEKVSHLLQDNLPK---------SVSTFQYDRFFEKKIDEKKND 268
ALAS1 Cavia porcellus         PNGLLKNFQDIMRKQRPERVSHLLQDNLPK---------SVSTFQYDRFFEKKIDEKKND 213
ALAS1 Chinchilla lanigera     PSGLLRNFQNIMRKQRPERVSHLLQDNLPK---------SVSTFQYDRFFEKKIDEKKND 213
ALAS1 Nannospalax galili      PNGLLKNFQDIMRKQRPERVSHLLQDNLPK---------SVSTFQYDRFFEKKIDEKKND 213
ALAS1 Castor canadensis       PSGLLKNFQDIMRKQRPEGVSHLLQDNLPK---------SVSTFQYDHFFEKKIDEKRND 213
ALAS1 Delphinapterus leucas   LSGLLKNFQDIMRKQRPEGVSHLLQDNLPK---------SVSTFQYDHFFEKKIDEKKND 237
ALAS1 Homo sapiens            PSGLLKNFQDIMQKQRPERVSHLLQDNLPK---------SVSTFQYDRFFEKKIDEKKND 213
ALAS1 Pan troglodytes         PSGLLKNFQDIMQKQRPERVSHLLQDNLPK---------SVSTFQYDRFFEKKIDEKKND 213
ALAS1 Macaca mulatta          PSGLLKNFQDIMQKQRPERVSHLLQDNLPK---------SVSTFQYDRFFEKKIDEKKND 213
ALAS1 Equus caballus          PSGLLKNFQDIMQKQRPERVSHLLQDNLPK---------SVSTFQYDRFFEKKIDEKKND 213
ALAS1 Odobenus rosmarus       TSGLLKNFQDIMRKQRPERVSHLLQDNLPK---------SVSTFQYDRFFEKKIDEKKND 280
ALAS1 Lontra canadensis       PGGLLKNFQDIMRKQRPERVSHLLQDNLPK---------SVSTFQYDHFFEKKIEEKKND 213
ALAS1 Hyaena hyaena           PSGLLKNFQDIMRKQRPERVSHLLQDNLPK---------SVSTFQYDRFFEKKIDEKKND 213
ALAS1 Acinonyx jubatus        PSGLLKNFQDIMRKQRPERVSHLLQDNLPK---------SISTFQYDRFFEKKIDEKKND 213
ALAS1 Panthera tigris         PSGLLKNFQDIMRKQRPERVSHLLQDNLPK---------SVSTFQYDRFFEKKIDEKKND 213
ALAS1 Panthera pardus         PSGLLKNFQDIMRKQRPERVSHLLQDNLPK---------SVSTFQYDRFFEKKIDEKKND 213
ALAS1 Lynx canadensis         PSGLLKNFQDIMRKQRPERVSHLLQDNLPK---------SVSTFQYDRFFEKKIDEKKND 213
ALAS1 Puma yagouaroundi       PSGLLKNFQDIMRKQRPERVSHLLQDNLPK---------SVSTFQYDRFFEKKIDEKKND 213
ALAS1 Puma concolor           PSGLLKNFQDIMRKQRPERVSHLLQDNLPK---------SVSTFQYDRFFEKKIDEKKND 213
ALAS1 Felis catus             PSGLLKNFQDIMRKQRPERVSHLLQDNLPK---------SVSTFQYDRFFEKKIDEKKND 213
ALAS1 Ailuropoda melanoleuca  PSGLLKNFQDIMRKQRPERVSHLLQDNLPK---------SVSTFQYDRFFEKKIDEKKND 213
ALAS1 Canis lupus dingo       PSGLLRNFQDIMRKQRPERVSHLLQDNLPK---------SVSTFQYDRFFEKKIDEKKND 272
ALAS2 Cavia porcellus         --KTFTSP--QDSEQTSGTVTHLSQKDMVG----------NHAFDYDQFFRNKIMEKKKD 159
```

```
ALAS2 Chinchilla lanigera       --KPFTGP--QDAEQTSGTLPHLIQNNMAG-----------IHAFDYDQFFRNKIMEKKQD 185
ALAS2 Rattus norvegicus         --QSVPRF--QDPEQTGGVPPLLIQNNMTG-----------SQAFGYDQFFRDKIMEKKQD 165
ALAS2 Mus musculus              --HSFPSF--QEPEQTEGAVPHLIQNNMTG-----------SQAFGYDQFFRDKIMEKKQD 159
ALAS2 Nannospalax galili        --KSFSSS--QDPEQMEGKLTQLVQNM------------IGNEAFGYDQFFKDKILEKKQD 158
ALAS2 Panthera tigris           --NPLSSL--PEPELISEKVTHLVQNNMVGEFDEVDIKRGNDVFGYDQFFRHKIMEKKQD 215
ALAS2 Panthera pardus           --NPLSSL--PEPELISEKVTHLVQNNMVG-----------NDVFGYDQFFRHKIMEKKQD 159
ALAS2 Puma concolor             --NPLSRL--PEPELISEKVTHLVQNNMV-----------GNDVFDYDQFFRHKIMEKKQD 159
ALAS2 Lynx canadensis           --NPLSSL--PEPELISEKVTHLVQNNMV-----------GNDVFDYDQFFRHKIMEKKQD 159
ALAS2 Puma yagouaroundi         --NPLSSL--PEPELISEKVTHLVQNNMV-----------GNDVFDYDQFFRHKIMEKKQD 159
ALAS2 Acinonyx jubatus          --NPLSTL--PEPELISEKVTHLVQNNMV-----------GNDVFDYDQFFRHKIMEKKQD 159
ALAS2 Felis catus               --NPLSSL--PEPELISEKVTHLVQNNMV-----------GNDVFDYDQFFRHKIMEKKQD 159
ALAS2 Lontra canadensis         --KPLSNL--QEPDL-ISEVTHQIQNNMVG-----------NHVFGYDQFFRHKIMEKKQD 158
ALAS2 Canis lupus dingo         --KPFSNP--QELELISEKVTHLVQNNMIG-----------NHVFGYDQFFRHKIMEKKQD 159
ALAS2 Ailuropoda melanoleuca    --KPFSNP--QEPELISEKVTQLVQNNMVG-----------NHVFGYDQFFRHKIMEKKQD 159
ALAS2 Odobenus rosmarus         --KPFSNP--QEPELISEKVTHLVQNNMIG-----------NHVFGYDQFFRHKIMEKKQD 159
ALAS2 Castor canadensis         --KSLPAS--QDPEQISEKLTQLIQDNMAG-----------IHAFGYDQFFRDKIMEKKLD 159
ALAS2 Ochotona curzoniae        --KQFSSP--QEPEQISGKVTHLIKNNMTG-----------NEAFGYDQFFRDKIMEKKQD 159
ALAS2 Oryctolagus cuniculus     --KPFSSP--QEPEQISGKVTHLIQNNMPG-----------NQAFGYDQFFRDKIMEKKQD 159
ALAS2 Homo sapiens              --KPFSGP--QEQEQISGKVTHLIQNNMPG-----------NYVFSYDQFFRDKIMEKKQD 159
ALAS2 Pan troglodytes           --KPFSGP--QEQEQISGKVTHLIQNNMPG-----------NYVFSYDQFFRDKIMEKKQD 159
ALAS2 Equus caballus            --EPFSSP--QEPEQTSEKVTHLIENNMAG-----------NHVFGYDQFFRDKIMEKKQD 159
ALAS2 Bos taurus                --KTFSSP--QEPEKNSEKVTHLIQNNMAG-----------DHVFGYDQFFRDKIMEKKQD 159
ALAS2 Delphinapterus leucas     --KPFFSP--QEPEKNSEKVTHLIQNNMAG-----------NHVFGYDQFFRNKIMEKKQD 191
ALAS2 Sarcophilus harrisii      --QLRGSG--DQKLTIENKITHLIKDNMSG-----------SHTFGYDAFFSHKIEEKKKD 213
ALAS2 Phascolarctos cinereus    --QFWGSG--GQKLALENKISHLIQDNMPG-----------SQAFAYDAFFSSKIEEKKRD 159
ALAS2 Vombatus ursinus          --QFWGSG--GQKLALENKISHLIQDNMPG-----------SHAFGYDAFFSRKIEEKKRD 159
                                                           :.             .* ** **  ** *** *
                                                      Catalytic Core  ------------>

ALAS1 Monodelphis domestica     HTYRVFKTVNRRAHIFPMADDYTDS-LVTKKQVSVWCSNDYLGMSRHPRVCGAVMETLKQ 271
ALAS1 Sarcophilus harrisii      HTYRVFKTVNRRAHIFPMADDYTDS-LVTKKQVSVWCSNDYLGMSRHPRVCGAVMETLKQ 271
ALAS1 Phascolarctos cinereus    HTYRVFKTVNRKAHIFPMADDYTDS-LVTKKQVSVWCSNDYLGMSRHPRVCGAVMETLKQ 271
ALAS1 Vombatus ursinus          HTYRVFKTVNRKAHIFPMADDYTDS-LVTKKQVSVWCSNDYLGMSRHPRVCGAVMETLKQ 271
ALAS1 Bos taurus                HSYRVFKTVNRKAQCFPMA DDYSDS-LISKQVSVWCSNDYLGMSRHPRVCGAVIDTLKQ 333
ALAS1 Meriones unguiculatus     HTYRVFKTVNRKAQTFPMADDYTDS-LITKKQVSVWCSNDYLGMSRHPRVCGAVMETVKQ 274
ALAS1 Rattus norvegicus         HTYRVFKTVNRRAQIFPMADDYTDS-LITKKQVSVWCSNDYLGMSRHPRVCGAVIETVKQ 274
ALAS1 Mus musculus              HTYRVFKTVNRRAQIFPMADDYTDS-LITKKQVSVWCSNDYLGMSRHPRVCGAVMETVKQ 274
ALAS1 Ochotona curzoniae        HTYRVFKTVNRRAHIFPMADDYSDS-LITKKQVSVWCSNDYLGMSRHPRVCGAVMDTLKQ 320
ALAS1 Oryctolagus cuniculus     HTYRVFKTVNRRAHIFPMADDYSDS-LITKKQVSVWCSNDYLGMSRHPRVCAAVMDTLKQ 327
ALAS1 Cavia porcellus           HTYRVFKTVNRRAHIFPMADDYSDS-LITKKQVSVWCSNDYLGMSRHPRVCAAVVETLKQ 272
ALAS1 Chinchilla lanigera       HTYRVFKTVNRRAQIFPMADDYSDS-LITKKQVSVWCSNDYLGMSRHPRVCAAVVETLKQ 272
ALAS1 Nannospalax galili        HTYRVFKTVNRRAHIFPMADDYSDS-LITKKQVSVWCSNDYLGMSRHPRVCGAVMETLKQ 272
ALAS1 Castor canadensis         HTYRVFKTVNRRAHIFPMADDYSDS-LITKKQVSVWCSNDYLGMSRHPRVCGAVVETLKQ 272
ALAS1 Delphinapterus leucas     HTYRVFKTVNRRAQFFPMADDYSDS-LVTKKQVSVWCSNDYLGMSCHPRVCGAVMDTLKQ 296
ALAS1 Homo sapiens              HTYRVFKTVNRRAHIFPMADDYSDS-LITKKQVSVWCSNDYLGMSRHPRVCGAVMDTLKQ 272
ALAS1 Pan troglodytes           HTYRVFKTVNRRAHIFPMADDYSDS-LITKKQVSVWCSNDYLGMSRHPRVCGAVMDTLKQ 272
ALAS1 Macaca mulatta            HTYRVFKTVNRRAHIFPMADDYSDS-LITKKQVSVWCSNDYLGMSRHPRVCGAVMDTLKQ 272
ALAS1 Equus caballus            HTYRVFKTVNRRAHIFPMADDYSDS-LITKKQVSVWCSNDYLGMSRHPRVCGAVMDTLKQ 272
ALAS1 Odobenus rosmarus         HTYRVFKTVNRRAHIFPMADDYSDS-LITKKQVSVWCSNDYLGMSRHPRVCGAVMDTLKQ 339
ALAS1 Lontra canadensis         HTYRVFKTVNRRAHIFPMADDYSDS-LITKKQVSVWCSNDYLGMSRHPRVCGAVMDTLKQ 272
ALAS1 Hyaena hyaena             HTYRVFKTVNRRAHIFPMADDYSDS-LITKKQVSVWCSNDYLGMSRHPRVCGAVMDTLKQ 272
ALAS1 Acinonyx jubatus          HTYRVFKTVNRRAHIFPMADDYSDS-LITKKQVSVWCSNDYLGMSRHPRVCGAVMDTLKQ 272
ALAS1 Panthera tigris           HTYRVFKTVNRRAHIFPMADDYSDS-LITKKQVSVWCSNDYLGMSRHPRVCGAVMDTLKQ 272
ALAS1 Panthera pardus           HTYRVFKTVNRRAHIFPMADDYSDS-LITKKQVSVWCSNDYLGMSRHPRVCGAVMDTLKQ 272
ALAS1 Lynx canadensis           HTYRVFKTVNRRAHIFPMADDYSDS-LITKKQVSVWCSNDYLGMSRHPRVCGAVMDTLKQ 272
ALAS1 Puma yagouaroundi         HTYRVFKTVNRRAHIFPMADDYSDS-LITKKQVSVWCSNDYLGMSRHPRVCGAVMDTLKQ 272
ALAS1 Puma concolor             HTYRVFKTVNRRAHIFPMADDYSDS-LITKKQVSVWCSNDYLGMSRHPRVCGAVMDTLKQ 272
ALAS1 Felis catus               HTYRVFKTVNRRAHIFPMADDYSDS-LITKKQVSVWCSNDYLGMSRHPRVCGAVMDTLKQ 272
ALAS1 Ailuropoda melanoleuca    HTYRVFKTVNRRAHIFPMADDYSDS-LITKKQVSVWCSNDYLGMSRHPRVCGAVMETLKQ 272
ALAS1 Canis lupus dingo         HTYRVFKTVNRRAHIFPMADDYSDS-LITKKQVSVWCSNDYLGMSRHPRVCGAVMDTLKQ 331
ALAS2 Cavia porcellus           HTYRVFKTVNRWADAYPFAQHFPEA-SVASKDVSVWCSNDYLGMSRHSRVLQATREILQL 218
ALAS2 Chinchilla lanigera       HTYRVFKTVNRWAEAYPFARHFPEA-SVAPKDVSVWCSNDYLGMSRHSRVLQATQEILHR 244
ALAS2 Rattus norvegicus         HTYRVFKTVNRWANAYPFAQHFSEA-SMDSKDVSVWCSNDYLGISRHPRVLQAIEETLKN 224
ALAS2 Mus musculus              HTYRVFKTVNRWANAYPFAQHFSEA-SMASKDVSVWCSNDYLGISRHPRVLQAIEETLKN 218
ALAS2 Nannospalax galili        HTYRVFKTVNRWADAYPFAQHFSET-STAPKDVSVWCSNDYLGISRHPRVLQAIHETLQH 217
ALAS2 Panthera tigris           HTYRVFKTVNRWADAYPFAQHFSEA-SVASKDVSVWCSNDYLGMSRHPRVLQATQETLQR 274
ALAS2 Panthera pardus           HTYRVFKTVNRWADAYPFAQHFSEA-SVASKDVSVWCSNDYLGMSRHPRVLQATQETLQR 218
ALAS2 Puma concolor             HTYRVFKTVNRWADAYPFAQHFSEA-SVASKDVSVWCSNDYLGMSRHPRVLQATQETLQR 218
ALAS2 Lynx canadensis           HTYRVFKTVNRWADAYPFAQHFSEA-SVASKDVSVWCSNDYLGMSRHPRVLQATQETLQR 218
ALAS2 Puma yagouaroundi         HSYRVFKTVNRWADAYPFAQHFSEA-SVASKDVSVWCSNDYLGMSRHPRVLQATQETLQR 218
ALAS2 Acinonyx jubatus          HTYRVFKTVNRWADAYPFAQHFSEA-SVASKDVSVWCSNDYLGMSRHPRVLQATQETLQR 218
```

```
ALAS2 Felis catus            HTYRVFKTVNRWADAYPFAQHFSEA-SVASKDVSVWCSNDYLGMSRHPRVLQATQETLQR 218
ALAS2 Lontra canadensis      HTYRVFKIVNRWADAYPFAQHFSEA-SMASKDVSVWCSNDYLGMSRHPQVLQATQETLQR 217
ALAS2 Canis lupus dingo      HTYRVFKTVNRWADAYPFAQHFSEA-SMASKDVSVWCSNDYLGMSRHPRVLQATQETLQR 218
ALAS2 Ailuropoda melanoleuca HTYRVFKTVNRWADAYPFAQHFSEA-SMASKDVSVWCSNDYLGMSRHPRVLQATKETLQR 218
ALAS2 Odobenus rosmarus      HTYRVFKTINRWADAYPFAQHFSEA-SMASKDVSVWCSNDYLGMSRHPRVLQATQETLQR 218
ALAS2 Castor canadensis      HTYRVFKTVNRWANAYPFAQHFSEA-FVASKDVSVWCSNDYLGMSRHPRVLQAIQETLQH 218
ALAS2 Ochotona curzoniae     HTYRVFKTVNRWADAYPFAQHFSEE-SVASKDVSVWCSNDYLGMSRHPRVLQATQDTLHR 218
ALAS2 Oryctolagus cuniculus  HTYRVFKTVNRWADAYPFAQHFSEV-SVASKDVSVWCSNDYLGMSRHPRVLQATQDTLNR 218
ALAS2 Homo sapiens           HTYRVFKTVNRWADAYPFAQHFSEA-SVASKDVSVWCSNDYLGMSRHPQVLQATQETLQR 218
ALAS2 Pan troglodytes        HTYRVFKTVNRWADAYPFAQHFSEA-SVASKDVSVWCSNDYLGMSRHPQVLQATQETLQR 218
ALAS2 Equus caballus         HTYRVFKTVNRWADAYPFAQHFFEA-SVASKDVSVWCSNDYLGMSRHPRVLQATQETLQR 218
ALAS2 Bos taurus             HTYRVFKTVNRWADAYPFAEHFFEA-SVASKDVSVWCSNDYLGMSRHPRVLQATQEILQR 218
ALAS2 Delphinapterus leucas  HTYRVFKTVNRWADAYPFAEHFFEA-SVASKDVSVWCSNDYLGMSRHPRVLQATQETLQR 250
ALAS2 Sarcophilus harrisii   HTYRVFKTVNRRADAYPFAQDFSEGESSGTKEVSIWCSNDYLGMSRHPSVLQATQDTLKQ 273
ALAS2 Phascolarctos cinereus HTYRVFKTVNRRADAYPLARDFSEGGSLTTKEVSIWCSNDYLGMSWHPSVLQATQDTLKQ 219
ALAS2 Vombatus ursinus       HSYRVFKTVNRRADAYPMAQDFSEGGSLTTKEVSIWCSNDYLGMSWHPSVLQATQDTLKQ 219
                             *:******:** *. :*:* .: :      *:**:********:* *  *  *  : ::

ALAS1 Monodelphis domestica  HGAGAGGTRNISGTSKFHVDLERELADLHGKDAALLFSSCFVANDSTLFTLAKMMPGCEI 331
ALAS1 Sarcophilus harrisii   HGAGAGGTRNISGTSKFHVDLEKELADLHGKDAALLFSSCFVANDSTLFTLAKMMPGCEI 331
ALAS1 Phascolarctos cinereus HGAGAGGTRNISGTSKFHVDLERELADLHGKDAALLFSSCFVANDSTLFTLAKMMPGCEI 331
ALAS1 Vombatus ursinus       HGAGAGGTRNISGTSKFHVDLERELADLHGKDAALLFSSCFVANDSTLFTLAKMMPGCEI 331
ALAS1 Bos taurus             HGTGAGGTRNISGTSKFHVDLEQELADLHGKDAALLFSSCFVANDSTLFTLAKMMPGCEI 393
ALAS1 Meriones unguiculatus  HGAGAGGTRNISGTSKFHVELEQALADLHGKDAALLFSSCFVANDSTLFTLAKMMPGCEI 334
ALAS1 Rattus norvegicus      HGAGAGGTRNISGTSKFHVELEQELADLHGKDAALLFSSCFVANDSTLFTLAKMMPGCEI 334
ALAS1 Mus musculus           HGAGAGGTRNISGTSKFHVELEQALADLHGKDAALLFSSCFVANDSTLFTLAKMMPGCEI 334
ALAS1 Ochotona curzoniae     HGAGAGGTRNISGTSKFHVDLERELADLHGKDAALLFSSCFVANDSTLFTLAKMMPDCEI 380
ALAS1 Oryctolagus cuniculus  HGAGAGGTRNISGTSKFHVDLERELADLHGKDAALLFSSCFVANDSTLFTLAKMMPGCEI 387
ALAS1 Cavia porcellus        HGAGAGGTRNISGTSKFHVDLEQELADLHGKDAALLFSSCFVANDSTLFTLAKMMPGCEI 332
ALAS1 Chinchilla lanigera    HGAGAGGTRNISGTSKFHVDLERELADLHGKDAALLFSSCFVANDSTLFTLAKMMPGCEI 332
ALAS1 Nannospalax galili     HGAGAGGTRNISGTSKFHVDLEQELADLHGKDAALLFSSCFVANDSTLFTLAKMMPGCEI 332
ALAS1 Castor canadensis      HGAGAGGTRNISGTSKFHVDLEQELADLHGKDAALLFSSCFVANDSTLFTLAKMMPGCEI 332
ALAS1 Delphinapterus leucas  HGTGAGGTRNISGTSKFHVDLEQELADLHGKDAALLFSSCFVANDSTLFTLAKMMPGCEI 356
ALAS1 Homo sapiens           HGAGAGGTRNISGTSKFHVDLERELADLHGKDAALLFSSCFVANDSTLFTLAKMMPGCEI 332
ALAS1 Pan troglodytes        HGAGAGGTRNISGTSKFHVDLERELADLHGKDAALLFSSCFVANDSTLFTLAKMMPGCEI 332
ALAS1 Macaca mulatta         HGAGAGGTRNISGTSKFHVDLEQELADLHGKDAALLFSSCFVANDSTLFTLAKMMPGCEI 332
ALAS1 Equus caballus         HGAGAGGTRNISGTSKFHVDLERELADLHGKDAALLFSSCFVANDSTLFTLAKMMPGCEI 332
ALAS1 Odobenus rosmarus      HGAGAGGTRNISGTSKFHVDLEQELADLHGKDAALLFSSCFVANDSTLFTLARMMPGCEI 399
ALAS1 Lontra canadensis      HGAGAGGTRNISGTSKFHVDLEQELADLHGKDAALLFSSCFVANDSTLFTLAKMMPGCEI 332
ALAS1 Hyaena hyaena          HGAGAGGTRNISGTSKFHVDLEQELADLHGKDAALLFSSCFVANDSTLFTLARMMPGCEI 332
ALAS1 Acinonyx jubatus       HGAGAGGTRNISGTSKFHVDLEQELADLHGKDAALLFSSCFVANDSTLFTLARMMPGCEI 332
ALAS1 Panthera tigris        HGAGAGGTRNISGTSKFHVDLEQELADLHGKDAALLFSSCFVANDSTLFTLARMMPGCEI 332
ALAS1 Panthera pardus        HGAGAGGTRNISGTSKFHVDLEQELADLHGKDAALLFSSCFVANDSTLFTLARMMPGCEI 332
ALAS1 Lynx canadensis        HGAGAGGTRNISGTSKFHVDLEQELADLHGKDAALLFSSCFVANDSTLFTLARMMPGCEI 332
ALAS1 Puma yagouaroundi      HGAGAGGTRNISGTSKFHVDLEQELADLHGKDAALLFSSCFVANDSTLFTLARMMPGCEI 332
ALAS1 Puma concolor          HGAGAGGTRNISGTSKFHVDLEQELADLHGKDAALLFSSCFVANDSTLFTLARMMPGCEI 332
ALAS1 Felis catus            HGAGAGGTRNISGTSKFHVDLEQELADLHGKDAALLFSSCFVANDSTLFTLARMMPGCEI 332
ALAS1 Ailuropoda melanoleuca HGAGAGGTRNISGTSKFHVDLEQELADLHGKDAALLFSSCFVANDSTLFTLARMMPGCEI 332
ALAS1 Canis lupus dingo      HGAGAGGTRNISGTSKFHVDLEQELADLHGKDAALLFSSCFVANDSTLFTLARMMPGCEI 391
ALAS2 Cavia porcellus        HGAGAGGTRNISGTSKFHVELEQELAELHQKDSALLFSSCFVANDSTLFTLAKILPGCEI 278
ALAS2 Chinchilla lanigera    HGAGAGGTRNISGTSKFHVELEQELAELHQKDSALLFSSCFVANDSTLFTLAKILPGCEI 304
ALAS2 Rattus norvegicus      HGAGAGGTRNISGTSKFHVELEQELAELHHKDSALLFSSCFVANDSTLFTLAKLLPGCEI 284
ALAS2 Mus musculus           HGAGAGGTRNISGTSKFHVELEQELAELHQKDSALLFSSCFVANDSTLFTLAKLLPGCEI 278
ALAS2 Nannospalax galili     HGAGAGGTRNISGTSKFHVELEQELAELHQKDSALLFSSCFVANDSTLFTLAKLLPGCEI 277
ALAS2 Panthera tigris        HGAGAGGTRNISGTSKFHVELEQELAELHQKDAALLFSSCFVANDSTLFTLAKILPGCKI 334
ALAS2 Panthera pardus        HGAGAGGTRNISGTSKFHVELEQELAELHQKDAALLFSSCFVANDSTLFTLAKILPGCKI 278
ALAS2 Puma concolor          HGAGAGGTRNISGTSKFHVELEQELAELHQKDAALLFSSCFVANDSTLFTLAKILPGCTI 278
ALAS2 Lynx canadensis        HGAGAGGTRNISGTSKFHVELEQELAELHQKDAALLFSSCFVANDSTLFTLAKILPGCTI 278
ALAS2 Puma yagouaroundi      HGAGAGGTRNISGTSKFHVELEQELAELHQKDAALLFSSCFVANDSTLFTLAKILPGCTI 278
ALAS2 Acinonyx jubatus       HGAGAGGTRNISGTSKFHVELEQELAELHQKDAALLFSSCFVANDSTLFTLAKILPGCTI 278
ALAS2 Felis catus            HGAGAGGTRNISGTSKFHVELEQELAELHQKDAALLFSSCFVANDSTLFTLAKILPGCTI 278
ALAS2 Lontra canadensis      HGAGAGGTRNISGTSKFHVELEQELAELHQKDAALLFSSCFVANDSTLFTLAKILPGCEI 277
ALAS2 Canis lupus dingo      HGAGAGGTRNISGTSKFHVELEQELAELHQKDAALLFSSCFVANDSTLFTLAKILPGCEI 278
ALAS2 Ailuropoda melanoleuca HGVGAGGTRNISGTSKFHVELEQELAELHQKDAALLFSSCFVANDSTLFTLAKILPGCEI 278
ALAS2 Odobenus rosmarus      HGAGAGGTRNISGTSKFHVELEQELAELHQKDAALLFSSCFVANDSTLFTLAKILPGCEI 278
ALAS2 Castor canadensis      HGAGAGGTRNISGTSKFHVELEQELAELHQKDSALLFSSCFVANDSTLFTLAKILPGCEI 278
ALAS2 Ochotona curzoniae     HGAGAGGTRNISGTSRFHVELEQELAELHQKDSALLFSSCFVANDSTLFTLAKILPGCEI 278
ALAS2 Oryctolagus cuniculus  HGAGAGGTRNISGTSKFHVELEQELAELHQKDSALLFSSCFVANDSTLFTLAKILPGCEI 278
ALAS2 Homo sapiens           HGAGAGGTRNISGTSKFHVELEQELAELHQKDSALLFSSCFVANDSTLFTLAKILPGCEI 278
ALAS2 Pan troglodytes        HGAGAGGTRNISGTSKFHVELEQELAELHQKDSALLFSSCFVANDSTLFTLAKILPGCEI 278
ALAS2 Equus caballus         HGAGAGGTRNISGTSKFHVELEQELAELHQKDSALLFSSCFVANDSTLFTLAKILPGCEI 278
```

```
ALAS2 Bos taurus                 HGAGAGGTRNISGTSKFHVELEQELAELHQKDSALLFSSCFVANDSTLFTLAKILPGCEI 278
ALAS2 Delphinapterus leucas      HGAGAGGTRNISGTSRFHVELEQELAELHQKDSALLFSSCFVANDSTLFTLAKILPGCEI 310
ALAS2 Sarcophilus harrisii       LGVGAGGTRNISGTSQFHVVLEQELADLHHKDAALLFSSCFVANDSTLFTLAKTLPGCEI 333
ALAS2 Phascolarctos cinereus     LGVGAGGTRNISGTSQFHVALEQELADLHYKDAALLFSSCFVANDSTLFTLARTLPGCEI 279
ALAS2 Vombatus ursinus           VGVGAGGTRNISGTSQFHVALERELADLHSKDAALLFSSCFVANDSTLFTLARTLPGCEM 279
                                  *.************:*** **: **:** **:*******************: :*.* :

ALAS1 Monodelphis domestica      YSDSGNHASMIQGIRNSRVPKYIFRHNDVNHLRELLKKSDPSVPKIVAFETVHSMDGAVC 391
ALAS1 Sarcophilus harrisii       YSDSGNHASMIQGIRNSRVPKYIFRHNDVSHLRELLKKSDPSIPKIVAFETVHSMDGAVC 391
ALAS1 Phascolarctos cinereus     YSDSGNHASMIQGIRNSRVPKYIFRHNDVSHLRELLKKSDPSVPKIVAFETVHSMDGAVC 391
ALAS1 Vombatus ursinus           YSDSGNHASMIQGIRNSRVPKYIFRHNDVSHLRELLKKSDPSVPKIVAFETVHSMDGAVC 391
ALAS1 Bos taurus                 YSDAGNHASMIQGIRNSGVPKYIFRHNDVSHLRELLQRSDPAVPKIVAFETVHSMDGAVC 453
ALAS1 Meriones unguiculatus      YSDAGNHASMIQGIRNSRVPKYIFRHNDVNHLRELLQRSDPSVPKIVAFETVHSMDGAVC 394
ALAS1 Rattus norvegicus          YSDSGNHASMIQGIRNSRVPKYIFRHNDVNHLRELLQRSDPSVPKIVAFETVHSMDGAVC 394
ALAS1 Mus musculus               YSDSGNHASMIQGIRNSRVPKYIFRHNDVNHLRELLQRSDPSVPKIVAFETVHSMDGAVC 394
ALAS1 Ochotona curzoniae         YSDAGNHASMIQGIRNSRVPKYIFRHNDVGHLRELLQRADPAVPKIVAFETVHSMDGAVC 440
ALAS1 Oryctolagus cuniculus      YSDSGNHASMIQGIRNSRVPKYIFRHNDAGHLRELLRRADPALPKIVAFETVHSMDGAVC 447
ALAS1 Cavia porcellus            YSDSGNHASMIQGIRNSRVPKYIFRHNDVHHLRELLQRSDPSVPKIVAFETVHSMDGAVC 392
ALAS1 Chinchilla lanigera        YSDSGNHASMIQGIRNSRVPKFIFRHNDVGHLRELLQRSDPSVPKIVAFETVHSMDGAVC 392
ALAS1 Nannospalax galili         YSDSGNHASMIQGIRNSRVPKYIFRHNDVSHLKELLQRSDPSVPKIVAFETVHSMDGAVC 392
ALAS1 Castor canadensis          YSDSGNHASMIQGIRNSRVPKYIFRHNDVSHLRELLQRADPSVPKIVAFETVHSMDGAVC 392
ALAS1 Delphinapterus leucas      YSDSGNHASMIQGIWNSRVPKYIYRHNDVDHLRELLQRSDPAVPKIVAFETVHSMDGAVC 416
ALAS1 Homo sapiens               YSDSGNHASMIQGIRNSRVPKYIFRHNDVSHLRELLQRSDPSVPKIVAFETVHSMDGAVC 392
ALAS1 Pan troglodytes            YSDSGNHASMIQGIRNSRVPKYIFRHNDVSHLRELLQRSDPSVPKIVAFETVHSMDGAVC 392
ALAS1 Macaca mulatta             YSDSGNHASMIQGIRNSRVPKYIFRHNDVSHLRELLQRSDPSVPKIVAFETVHSMDGAVC 392
ALAS1 Equus caballus             YSDSGNHASMIQGIRNSRVPKYIFRHNDVSHLRELLQRSDPSVPKIVAFETVHSMDGAVC 392
ALAS1 Odobenus rosmarus          YSDSGNHASMIQGIRNSRVPKYIFRHNDVSHLRELLQRSDPAVPKIVAFETVHSMDGAVC 459
ALAS1 Lontra canadensis          YSDSGNHASMIQGIRNSRVPKYIFRHNDVSHLRELLQRSDPSVPKIVAFETVHSMDGAVC 392
ALAS1 Hyaena hyaena              YSDSGNHASMIQGIRNSRVPKYIFRHNDVSHLRELLQRSDPSVPKIVAFETVHSMDGAVC 392
ALAS1 Acinonyx jubatus           YSDSGNHASMIQGIRNSRVPKYIFRHNDVSHLRELLQRSDPSVPKIVAFETVHSMDGAVC 392
ALAS1 Panthera tigris            YSDSGNHASMIQGIRNSRVPKYIFRHNDVSHLRELLQRSDPSVPKIVAFETVHSMDGAVC 392
ALAS1 Panthera pardus            YSDSGNHASMIQGIRNSRVPKYIFRHNDVSHLRELLQRSDPSVPKIVAFETVHSMDGAVC 392
ALAS1 Lynx canadensis            YSDSGNHASMIQGIRNSRVPKYIFRHNDVSHLRELLQRSDPSVPKIVAFETVHSMDGAVC 392
ALAS1 Puma yagouaroundi          YSDSGNHASMIQGIRNSRVPKYIFRHNDVSHLRELLQRSDPSVPKIVAFETVHSMDGAVC 392
ALAS1 Puma concolor              YSDSGNHASMIQGIRNSRVPKYIFRHNDVSHLRELLQRSDPSVPKIVAFETVHSMDGAVC 392
ALAS1 Felis catus                YSDSGNHASMIQGIRNSRVPKYIFRHNDVSHLRELLQRSDPSVPKIVAFETVHSMDGAVC 392
ALAS1 Ailuropoda melanoleuca     YSDSGNHASMIQGIRNSRVPKYIFRHNDVSHLRELLQRSDPSVPKIVAFETVHSMDGAVC 392
ALAS1 Canis lupus dingo          YSDSGNHASMIQGIRNSRVPKYIFRHNDVSHLRELLQRSDPSVPKIVAFETVHSMDGAVC 451
ALAS2 Cavia porcellus            YSDAGNHASMIQGIRNSGAAKFVFKHNDPDHLEKLLEKSNPKTPKIVAFETVHSMDGAIC 338
ALAS2 Chinchilla lanigera        YSDAGNHASMIQGIRNSGAAKFVFRHNDPDHLKKLLEKSNPKTPKIVAFETVHSMDGAIC 364
ALAS2 Rattus norvegicus          YSDAGNHASMIQGIRNSGAVKFVFRHNDPGHLKKLLEKSDPKTPKIVAFETVHSMDGAIC 344
ALAS2 Mus musculus               YSDAGNHASMIQGIRNSGAAKFVFRHNDPGHLKKLLEKSDPKTPKIVAFETVHSMDGAIC 338
ALAS2 Nannospalax galili         YSDAGNHASMIQGIRNSGVAKFVFRHNDPDHLKKLLEKSNPKTPKIVAFETVHSMDGAIC 337
ALAS2 Panthera tigris            YSDAGNHASMIQGIRNSGAAKFVFRHNDPDHLKKLLKESNSKIPKIVAFETVHSMDGAIC 394
ALAS2 Panthera pardus            YSDAGNHASMIQGIRNSGAAKFVFRHNDPDHLKKLLKESNSKIPKIVAFETVHSMDGAIC 338
ALAS2 Puma concolor              YSDAGNHASMIQGIRNSGAAKFVFRHNDPDHLKKLLKESNPKIPKIVAFETVHSMDGAIC 338
ALAS2 Lynx canadensis            YSDAGNHASMIQGIRNSGAAKFVFRHNDPDHLKKLLKESNPKIPKIVAFETVHSMDGAIC 338
ALAS2 Puma yagouaroundi          YSDAGNHASMIQGIRNSGAAKFVFRHNDPDHLKKLLKESNPKIPKIVAFETVHSMDGAIC 338
ALAS2 Acinonyx jubatus           YSDAGNHASMIQGIRNSGAAKFVFRHNDPDHLKKLLKESNPKIPKIVAFETVHSMDGAIC 338
ALAS2 Felis catus                YSDAGNHASMIQGIRNSGAAKFVFRHNDPDHLKKLLKESNPKIPKIVAFETVHSMDGAIC 338
ALAS2 Lontra canadensis          YSDAGNHASMIQGIRNSGAAKFVFRHNDPDHLKKLLKESNLKTPKIVAFETVHSMDGAIC 337
ALAS2 Canis lupus dingo          YSDAGNHASMIQGIRNSGAAKFVFRHNDPDHLKKLLKESNTRTPKIVAFETVHSMDGAIC 338
ALAS2 Ailuropoda melanoleuca     YSDAGNHASMIQGIRNSGAAKFVFRHNDPDHLKKLLKESNPKTPKIVAFETVHSMDGAIC 338
ALAS2 Odobenus rosmarus          YSDAGNHASMIQGIRNSGAAKFVFRHNDPDHLKKLLKESNPRTPKIVAFETVHSMDGAIC 338
ALAS2 Castor canadensis          YSDAGNHASMIQGIRNSGAAKFVFRHNDPDHLKKLLEKSNRKTPKIVAFETVHSMDGAIC 338
ALAS2 Ochotona curzoniae         YSDAGNHASMIQGIRNSGAAKFVFRHNDPDHLKKLLEKSNPKTPKIVAFETVHSMDGAIC 338
ALAS2 Oryctolagus cuniculus      YSDAGNHASMIQGIRNSGAAKFVFRHNDPDHLKKLLEKSNPKTPKIVAFETVHSMDGAIC 338
ALAS2 Homo sapiens               YSDAGNHASMIQGIRNSGAAKFVFRHNDPDHLKKLLEKSNPKIPKIVAFETVHSMDGAIC 338
ALAS2 Pan troglodytes            YSDAGNHASMIQGIRNSGAAKFVFRHNDPDHLKKLLEKSNPKIPKIVAFETVHSMDGAIC 338
ALAS2 Equus caballus             YSDAGNHASMIQGIRNSGAAKFVFRHNDPDHLKKLLEKSNPETPKIVAFETVHSMDGAIC 338
ALAS2 Bos taurus                 YSDAGNHASMIQGIRNSGAAKFVFRHNDPDHLKKLLKKSNPETPKIVAFETVHSMDGAIC 338
ALAS2 Delphinapterus leucas      YSDAGNHASMIQGIRNSGAAKFVFRHNDPDHLKKLLKKSNPETPKIVAFETVHSMDGAIC 370
ALAS2 Sarcophilus harrisii       YSDAGNHASMIQGIRNSGVPKFVFRHNDPAHLEELLSKAEPLTPKIVAFETVHSMDGAIC 393
ALAS2 Phascolarctos cinereus     YSDAGNHASMIQGIRNSGVPKFVFRHNDPEHLEELLSKADPLTPKIVAFETVHSMDGAIC 339
ALAS2 Vombatus ursinus           YSDAGNHASMIQGIRNSGVPKFVFRHNDPEHLEELLSKADPLTPKIVAFETVHSMDGAIC 339
                                 ***:********** **  . *::::***  **.:** .::   ***************:*

ALAS1 Monodelphis domestica      PLEELCDVAHEYGAITFVDEVHAVGLYGARGGGIGDRDGIMHKMDIISGTLGKAFGCVGG 451
ALAS1 Sarcophilus harrisii       PLEELCDVAHEYGAITFVDEVHAVGLYGARGGGIGDRDGIMHKMDIISGTLGKAFGCVGG 451
ALAS1 Phascolarctos cinereus     PLEELCDVAHEYGAITFVDEVHAVGLYGARGGGIGDRDGIMHKMDIISGTLGKAFGCVGG 451
ALAS1 Vombatus ursinus           PLEELCDVAHEYGAITFVDEVHAVGLYGARGGGIGDRDGVMHKMDIISGTLGKAFGCVGG 451
```

```
ALAS1 Bos taurus                  PLEELCDVAHEFGAITFVDEVHAVGLYGLQGGGIGDRDGVMPKMDIISGTLGKAIGCVGG 513
ALAS1 Meriones unguiculatus       PLEELCDVAHEFGAITFVDEVHAVGLYGARGGGIGDRDGVMPKMDIISGTLGKAFGCVGG 454
ALAS1 Rattus norvegicus           PLEELCDVAHEFGAITFVDEVHAVGLYGASGGGIGDRDGVMPKMDIISGTLGKAFGCVGG 454
ALAS1 Mus musculus                PLEELCDVAHEFGAITFVDEVHAVGLYGARGGGIGDRDGVMPKMDIISGTLGKAFGCVGG 454
ALAS1 Ochotona curzoniae          PLQELCDVAHEFGAITFVDEVHAVGLYGAHGGGIGDRDGVMPKMDIISGTLGKAFGCVGG 500
ALAS1 Oryctolagus cuniculus       PLEELCDVAHEFGAITFVDEVHAVGLYGARGGGIGDRDGVMPKMDIISGTLGKAFGCVGG 507
ALAS1 Cavia porcellus             PLEELCDVAHEFGAITFVDEVHAVGLYGAQGGGIGDRDGVMPKMDIISGTLGKAFGCVGG 452
ALAS1 Chinchilla lanigera         PLEELCDVAHEFGAITFVDEVHAVGLYGAHGGGIGDRDGVMPKMDIISGTLGKAFGCVGG 452
ALAS1 Nannospalax galili          PLEELCDVAHEFGAITFVDEVHAVGLYGARGGGIGDRDGVMPKMDIISGTLGKAFGCVGG 452
ALAS1 Castor canadensis           PLEELCDVAHEFGAITFVDEVHAVGLYGARGGGIGDRDGVMPKMDIISGTLGKAFGCVGG 452
ALAS1 Delphinapterus leucas       PLEELCDVAHEFGAITFVDEVHAVGLYGAQGGGIGDRDGVMPKMDIISGTLGKAFGCVGG 476
ALAS1 Homo sapiens                PLEELCDVAHEFGAITFVDEVHAVGLYGARGGGIGDRDGVMPKMDIISGTLGKAFGCVGG 452
ALAS1 Pan troglodytes             PLEELCDVAHEFGAITFVDEVHAVGLYGARGGGIGDRDGVMPKMDIISGTLGKAFGCVGG 452
ALAS1 Macaca mulatta              PLEELCDVAHEFGAITFVDEVHAVGLYGARGGGIGDRDGVMPKMDIISGTLGKAFGCVGG 452
ALAS1 Equus caballus              PLEELCDVAHEFGAITFVDEVHAVGLYGARGGGIGDRDGVMPKMDIISGTLGKAFGCVGG 452
ALAS1 Odobenus rosmarus           PLEELCDVAHEFGAITFVDEVHAVGLYGAQGGGIGDRDGVMPKMDIISGTLGKAFGCVGG 519
ALAS1 Lontra canadensis           PLEELCDVAHEFGAITFVDEVHAVGLYGARGGGIGDRDGVMPKMDIISGTLGKAFGCVGG 452
ALAS1 Hyaena hyaena               PLEELCDVAHEFGAITFVDEVHAVGLYGARGGGIGDRDGVMPKMDIISGTLGKAFGCVGG 452
ALAS1 Acinonyx jubatus            PLEELCDVAHEFGAITFVDEVHAVGLYGARGGGIGDRDGVMPKMDIISGTLGKAFGCVGG 452
ALAS1 Panthera tigris             PLEELCDVAHEFGAITFVDEVHAVGLYGARGGGIGDRDGVMPKMDIISGTLGKAFGCVGG 452
ALAS1 Panthera pardus             PLEELCDVAHEFGAITFVDEVHAVGLYGARGGGIGDRDGVMPKMDIISGTLGKAFGCVGG 452
ALAS1 Lynx canadensis             PLEELCDVAHEFGAITFVDEVHAVGLYGARGGGIGDRDGVMPKMDIISGTLGKAFGCVGG 452
ALAS1 Puma yagouaroundi           PLEELCDVAHEFGAITFVDEVHAVGLYGARGGGIGDRDGVMPKMDIISGTLGKAFGCVGG 452
ALAS1 Puma concolor               PLEELCDVAHEFGAITFVDEVHAVGLYGARGGGIGDRDGVMPKMDIISGTLGKAFGCVGG 452
ALAS1 Felis catus                 PLEELCDVAHEFGAITFVDEVHAVGLYGARGGGIGDRDGVMPKMDIISGTLGKAFGCVGG 452
ALAS1 Ailuropoda melanoleuca      PLEELCDVAHEFGAITFVDEVHAVGLYGARGGGIGDRDGVMPKMDIISGTLGKAFGCVGG 452
ALAS1 Canis lupus dingo           PLEELCDVAHEFGAITFVDEVHAVGLYGARGGGIGDRDGVMPKMDIISGTLGKAFGCVGG 511
ALAS2 Cavia porcellus             PLEELCDVAHHYGALTFVDEVHAVGLYGSRGAGIGDEDGVMHKMDIISGTLGKAFGCVGG 398
ALAS2 Chinchilla lanigera         PLEELCDVAHHYGALTFVDEVHAVGLYGSHGAGIGDEDGVMHKMDIISGTLGKAFGCVGG 424
ALAS2 Rattus norvegicus           PLEELCDVAHQYGALTFVDEVHAVGLYGTRGAGIGERDGIMHKLDIISGTLGKAFGCVGG 404
ALAS2 Mus musculus                PLEELCDVAHQYGALTFVDEVHAVGLYGARGAGIGERDGIMHKLDIISGTLGKAFGCVGG 398
ALAS2 Nannospalax galili          PLEELCDVAHQYGALTFVDEVHAVGLYGTRGAGIGERDGIMYKLDIISGTLGKAFGCVGG 397
ALAS2 Panthera tigris             PLEELCDVAHQYGALTFVDEVHAVGLYGSRGAGIGERDGVMHKIDIVSGTLGKAFGCVGG 454
ALAS2 Panthera pardus             PLEELCDVAHQYGALTFVDEVHAVGLYGSRGAGIGERDGVMHKIDIVSGTLGKAFGCVGG 398
ALAS2 Puma concolor               PLEELCDVAHQYGALTFVDEVHAVGLYGSRGAGIGERDGVMHKIDIVSGTLGKAFGCVGG 398
ALAS2 Lynx canadensis             PLEELCDVAHQYGALTFVDEVHAVGLYGSRGAGIGERDGVMHKIDIVSGTLGKAFGCVGG 398
ALAS2 Puma yagouaroundi           PLEELCDVAHQYGALTFVDEVHAVGLYGSRGAGIGERDGVMHKIDIVSGTLGKAFGCVGG 398
ALAS2 Acinonyx jubatus            PLEELCDVAHQYGALTFVDEVHAVGLYGSRGAGIGERDGVMHKIDIVSGTLGKAFGCVGG 398
ALAS2 Felis catus                 PLEELCDVAHQYGALTFVDEVHAVGLYGSRGAGIGERDGVMHKIDIVSGTLGKAFGCVGG 398
ALAS2 Lontra canadensis           PLEELCDVAHQYGALTFVDEVHAVGLYGSRGAGIGERDGIMYKIDIVSGTLGKAFGCVGG 397
ALAS2 Canis lupus dingo           PLEELCDVAHQYGALTFVDEVHAVGLYGPQGAGIGERDGVMHKIDIVSGTLGKAFGCVGG 398
ALAS2 Ailuropoda melanoleuca      PLEELCDVAHQYGALTFVDEVHAVGLYGSRGAGIGDEDGVMHKMDIISGTLGKAFGCVGG 398
ALAS2 Odobenus rosmarus           PLEELCDVAHQYGALTFVDEVHAVGLYGSRGAGIGERDGIMYKIDIVSGTLGKAFGCVGG 398
ALAS2 Castor canadensis           PLEELCDVAHQYGALTFVDEVHAVGLYGSRGAGIGERDGIMHKIDIISGTLGKAFGCVGG 398
ALAS2 Ochotona curzoniae          PLEELCDIAHEHGALTFVDEVHAVGLYGSRGAGIGERDGIMHKIDIISGTLGKAFGCVGG 398
ALAS2 Oryctolagus cuniculus       PLEELCDVAHQHGALTFVDEVHAVGLYGSRGAGIGERDGIMHKIDIISGTLGKAFGCVGG 398
ALAS2 Homo sapiens                PLEELCDVSHQYGALTFVDEVHAVGLYGSRGAGIGERDGIMHKIDIISGTLGKAFGCVGG 398
ALAS2 Pan troglodytes             PLEELCDVSHQYGALTFVDEVHAVGLYGSRGAGIGERDGIMHKIDIISGTLGKAFGCVGG 398
ALAS2 Equus caballus              PLEELCDVAHQYGALTFVDEVHAVGLYGSRGAGIGERDGIMHKMDIISGTLGKAFGCVGG 398
ALAS2 Bos taurus                  PLEELCDVAHQYGALTFVDEVHAVGLYGSRGAGIGERDGIMHKIDIISGTLGKAFGCVGG 398
ALAS2 Delphinapterus leucas       PLEELCDVAHQYGALTFVDEVHAVGLYGSRGAGIGERDGIMHKIDIISGTLGKAFGCVGG 430
ALAS2 Sarcophilus harrisii        PLEEMCDIAHKYGALTFVDEVHAVGLYGSRGAGIGERDGVMHKIDIVSGTLGKAFGCIGG 453
ALAS2 Phascolarctos cinereus      PLEEMCDIAHKYGALTFVDEVHAVGLYGARGAGIGERDGVMHKIDIISGTLGKAFGCVGG 399
ALAS2 Vombatus ursinus            PLEEMCDVAHKYGALTFVDEVHAVGLYGARGAGIGERDGVMHKIDIVSGTLGKAFGCVGG 399
                                  **:*:**::*..**:*************  *.***:***:* *:**:******:**:**

ALAS1 Monodelphis domestica       YVASTNALIDTVRSYAAGFIFTTSLPPMLLAGALESVRILKSAEGQALRRQHQRNVKLMR 511
ALAS1 Sarcophilus harrisii        YIASTSSLIDTVRSYAAGFIFTTSLPPMLLAGALESVRILKSAEGQALRRQHQRNVKLMR 511
ALAS1 Phascolarctos cinereus      YIASTSSLIDTVRSYAAGFIFTTSLPPMLLAGALESVRILKSAEGRALRRQHQRNVKLMR 511
ALAS1 Vombatus ursinus            YIASTGSLIDTVRSYAAGFIFTTSLPPMLLAGALESVRILKSAEGRALRRQHQRNVKLMR 511
ALAS1 Bos taurus                  YIASTSSLIDTVRSYAAGFIFTTSLPPMLLAGALESVRILRSTEGRTLRRQHQRNVKLMR 573
ALAS1 Meriones unguiculatus       YIASTRLLIDTVRSYAAGFIFTTSLPPMLLAGALESVRILKSNEGRALRRQHQRNVKLMR 514
ALAS1 Rattus norvegicus           YIASTSLLIDTVRSYAAGFIFTTSLPPMLLAGALESVRILKSNEGRALRRQHQRNVKLMR 514
ALAS1 Mus musculus                YIASTSLLIDTVRSYAAGFIFTTSLPPMLLAGALESVRILKSSEGRALRRQHQRNVKLLR 514
ALAS1 Ochotona curzoniae          YIASTSSLIDTVRSYAAGFIFTTSLPPMLLAGALESVRVLKSAEGRALRRQHQRNVKLMR 560
ALAS1 Oryctolagus cuniculus       YIASTSSLIDTVRSYAAGFIFTTSLPPMLLAGALESVRILKSAEGRALRRQHQRNVKLMR 567
ALAS1 Cavia porcellus             YIASTSSLIDTVRSYAAGFIFTTSLPPMLLAGALESVRILKSAEGRALRRQHQRNVKLMR 512
ALAS1 Chinchilla lanigera         YIASTSSLIDTVRSYAAGFIFTTSLPPMLLAGALESVRILKSAEGRALRRQHQRNVKLMR 512
ALAS1 Nannospalax galili          YIASTNSLIDTVRSYAAGFIFTTSLPPMLLAGALESVRILKSAEGRALRRQHQRNVKLMR 512
ALAS1 Castor canadensis           YIASTSSLVDTVRSYAAGFIFTTSLPPMLLAGALESVRILKSAEGQALRRQHQRNVKLMR 512
ALAS1 Delphinapterus leucas       YIASTSSLIDTIRSYAAGFIFTTSLPPMLLAGALESVRILKSTEGRVLRRQHQRNVKLMR 536
```

```
ALAS1  Homo sapiens              YIASTSSLIDTVRSYAAGFIFTTSLPPMLLAGALESVRILKSAEGRVLRRQHQRNVKLMR 512
ALAS1  Pan troglodytes           YIASTSSLIDTVRSYAAGFIFTTSLPPMLLAGALESVRILKSAEGRVLRRQHQRNVKLMR 512
ALAS1  Macaca mulatta            YIASTSSLIDTVRSYAAGFIFTTSLPPMLLAGALESVRILKSAEGRVLRRQHQRNVKLMR 512
ALAS1  Equus caballus            YIASTSSLIDTVRSYAAGFIFTTSLPPMLLAGALESVRILKSAEGRALRRQHQRNVKLMR 512
ALAS1  Odobenus rosmarus         YIASTSSLIDTVRSYAAGFIFTTSLPPMLLAGALESVRILKSAEGRALRRQHQRNVKLMR 579
ALAS1  Lontra canadensis         YIASTSSLIDTVRSYAAGFIFTTSLPPMLLAGALESVRILKSAEGRALRRQHQRNVKLMR 512
ALAS1  Hyaena hyaena             YIASTSSLIDTVRSYAAGFIFTTSLPPMLLAGALESVRILKSAEGRALRRQHQRNVKLMR 512
ALAS1  Acinonyx jubatus          YIASTSSLIDTVRSYAAGFIFTTSLPPMLLAGALESVRILKSAEGRALRRQHQRNVKLMR 512
ALAS1  Panthera tigris           YIASTSSLIDTVRSYAAGFIFTTSLPPMLLAGALESVRILKSAEGRALRRQHQRNVKLMR 512
ALAS1  Panthera pardus           YIASTSSLIDTVRSYAAGFIFTTSLPPMLLAGALESVRILKSAEGRALRRQHQRNVKLMR 512
ALAS1  Lynx canadensis           YIASTSSLIDTVRSYAAGFIFTTSLPPMLLAGALESVRILKSAEGRALRRQHQRNVKLMR 512
ALAS1  Puma yagouaroundi         YIASTSSLIDTVRSYAAGFIFTTSLPPMLLAGALESVRILKSAEGRALRRQHQRNVKLMR 512
ALAS1  Puma concolor             YIASTSSLIDTVRSYAAGFIFTTSLPPMLLAGALESVRILKSAEGRALRRQHQRNVKLMR 512
ALAS1  Felis catus               YIASTSSLIDTVRSYAAGFIFTTSLPPMLLAGALESVRILKSAEGRALRRQHQRNVKLMR 512
ALAS1  Ailuropoda melanoleuca    YIASTSSLIDTVRSYAAGFIFTTSLPPMLLAGALESVRILKSAEGRALRRQHQRNVKLMR 512
ALAS1  Canis lupus dingo         YIASTSSLIDTVRSYAAGFIFTTSLPPMLLAGALESVRILKSAEGRALRRQHQRNVKLMR 571
ALAS2  Cavia porcellus           YIASTQHLVDMVRSYAAGFIFTTSLPPMVLSGALESVRVLKGEEGQALRRAHQRNVKHMR 458
ALAS2  Chinchilla lanigera       YIASTYHLVDMVRSYAAGFIFTTSLPPMVLSGALESVRVLKGEEGQALRRAHQRNVKHMR 484
ALAS2  Rattus norvegicus         YIASTRDLVDMVRSYAAGFIFTTSLPPMVLSGALESVRLLKGEEGQALRRAHQRNVKHMR 464
ALAS2  Mus musculus              YIASTRDLVDMVRSYAAGFIFTTSLPPMVLSGALESVRLLKGEEGQALRRAHQRNVKHMR 458
ALAS2  Nannospalax galili        YIASTRDLVDMVRSYAAGFIFTTSLPPMVLSGALESVRLLKGEEGQVLRRAHQRNVKHMR 457
ALAS2  Panthera tigris           YIASTSDLVDMIRSYAAGFIFTTSLPPMVLSGALESVQLLKGEEGQALRRAHQRNVKHMR 514
ALAS2  Panthera pardus           YIASTSDLVDMIRSYAAGFIFTTSLPPMVLSGALESVQLLKGEEGQALRRAHQRNVKHMR 458
ALAS2  Puma concolor             YIASTSDLVDMIRSYAAGFIFTTSLPPMVLSGALESVQLLKGEEGQALRRAHQRNVKHMR 458
ALAS2  Lynx canadensis           YIASTSDLVDMIRSYAAGFIFTTSLPPMVLSGALESVQLLKGEEGQALRRAHQRNVKHMR 458
ALAS2  Puma yagouaroundi         YIASTSDLVDMIRSYAAGFIFTTSLPPMVLSGALESVQLLKGEEGQALRRAHQRNVKHMR 458
ALAS2  Acinonyx jubatus          YIASTSDLVDMIRSYAAGFIFTTSLPPMVLSGALESVQLLKGEEGQALRRAHQRNVKHMR 458
ALAS2  Felis catus               YIASTSDLVDMIRSYAAGFIFTTSLPPMVLSGALESVQLLKGEEGQALRRAHQRNVKHMR 458
ALAS2  Lontra canadensis         YIASTHDLVDMVRSYAAGFIFTTSLPPMVLSGALESVRLLKGEEGQALRRAHQRNVKHMR 457
ALAS2  Canis lupus dingo         YIASTRDLVDMVRSYAAGFIFTTSLPPMVLSGALESVRLLKGEEGQALRRAHQRNVKHMR 458
ALAS2  Ailuropoda melanoleuca    YIASTRDLVDMVRSYAAGFIFTTSLPPMVLSGALESVRLLKAEEGQALRRAHQRNVKHMR 458
ALAS2  Odobenus rosmarus         YIASTRDLVDMVRSYAAGFIFTTSLPPMVLSGALESVRLLKGEEGQALRRAHQRNVKHMR 458
ALAS2  Castor canadensis         YIASTRDLVDMVRSYAAGFIFTTSLPPMVLSGALESVRLLKGEEGQALRRAHQRNVKHMR 458
ALAS2  Ochotona curzoniae        YIASTRDLVDTVRSYAAGFIFTTSLPPMVLSGALESVRLLKGEEGQALRRAHQRNVKHMR 458
ALAS2  Oryctolagus cuniculus     YIASTRDLVDTVRSYAAGFIFTTSLPPMVLSGALESVRLLKGEEGQALRRAHQRNVKHMR 458
ALAS2  Homo sapiens              YIASTRDLVDMVRSYAAGFIFTTSLPPMVLSGALESVRLLKGEEGQALRRAHQRNVKHMR 458
ALAS2  Pan troglodytes           YIASTRDLVDMVRSYAAGFIFTTSLPPMVLSGALESVRLLKGEEGQALRRAHQRNVKHMR 458
ALAS2  Equus caballus            YIASTRDLVDMVRSYAAGFIFTTSLPPMVLSGALESVRLLKGEEGQALRRAHQRNVKHMR 458
ALAS2  Bos taurus                YIASTRDLVDMVRSYAAGFIFTTSLPPMVLSGALESVRLLKGEEGQALRRAHQRNVKHMR 458
ALAS2  Delphinapterus leucas     YIASTRDLVDMVRSYAAGFIFTTSLPPMVLSGALESVRLLKGEEGQALRRAHQRNVKHMR 490
ALAS2  Sarcophilus harrisii      YIASTRDLVDTVRSYAAGFIFTTSLPPMVLAGALASVRILKGKEGQALRRAHQRNVRHMR 513
ALAS2  Phascolarctos cinereus    YIASTRDLVDTMRSYAAGFIFTTSLPPMVLAGALASVRILKGEEGQALRRAHQRNVRHMR 459
ALAS2  Vombatus ursinus          YIASTRDLVDTVRSYAAGFIFTTSLPPMVLAGALASVRILKGEEGQVLRRAHQRNVRHMR 459
                                 *:***   *:*  :****************:*:***  **::*:.  **:.***  *****:  :*

ALAS1  Monodelphis domestica     QMLMDAGLPVVHCPSHIIPVRVADAAKNTEVCDELMSKHNIYVQAINYPTVPRGEELLRI 571
ALAS1  Sarcophilus harrisii      QMLMDAGLPVVHCPSHIIPVRVADAAKNTEVCDELMSKHNIYVQAINYPTVPRGEELLRI 571
ALAS1  Phascolarctos cinereus    QMLMDXGLPVVHCPSHIIPVRVADAAKNTEVCDELMSKHNIYVQAINYPTVPRGEELLRI 571
ALAS1  Vombatus ursinus          QMLMDAGLPVVHCPSHIIPVRVADAAKNTEVCDELMSKHNIYVQAINYPTVPRGEELLRI 571
ALAS1  Bos taurus                QMLMDAGLPVVHCPSHIIPVRVADAAKNTEVCDELMTRHNIYVQAINYPTVPRGEELLRI 633
ALAS1  Meriones unguiculatus     QMLMDAGLPVIHCPSHIIPVRVADAAKNTEICDELMTRHNIYVQAINYPTVPRGEELLRI 574
ALAS1  Rattus norvegicus         QMLMDAGLPVIHCPSHIIPVRVADAAKNTEICDELMTRHNIYVQAINYPTVPRGEELLRI 574
ALAS1  Mus musculus              QMLMDAGLPVIHCPSHIIPVRVADAAKNTEICDELMTRHNIYVQAINYPTVPRGEELLRI 574
ALAS1  Ochotona curzoniae        QMLMDAGLPVVHCPSHIIPVRVADAAKNTAICDELMSRHNIYVQAINYPTVPRGEELLRI 620
ALAS1  Oryctolagus cuniculus     QMLMDAGLPVVHCPSHIIPVRVADAAKNTAVCDELMSRHNIYVQAINYPTVPRGEELLRI 627
ALAS1  Cavia porcellus           QMLMDAGLPVVHCPSHIIPVRVADAAKNTQVCDELMSRHNIYVQAINYPTVPRGEELLRI 572
ALAS1  Chinchilla lanigera       QMLMDAGLPVVHCPSHIIPVRVADAAKNTQVCDELMSRHNIYVQAINYPTVPRGEELLRI 572
ALAS1  Nannospalax galili        QMLMDAGLPVIHCPSHIIPVRVANAAKNTEICDELMSKHNIYVQAINYPTVPRGEELLRI 572
ALAS1  Castor canadensis         QMLMDAGLPVIHCPSHIIPVRVADAAKNTEVCDELMSRHNIYVQAINYPTVPRGEELLRI 572
ALAS1  Delphinapterus leucas     QMLMDASLPVVHCPSHIIPVRVADAAKNTEVCNELMSRHNIYVQAINYPTVRRGEELLRI 596
ALAS1  Homo sapiens              QMLMDAGLPVVHCPSHIIPVRVADAAKNTEVCDELMSRHNIYVQAINYPTVPRGEELLRI 572
ALAS1  Pan troglodytes           QMLMDAGLPVVHCPSHIIPVRVADAAKNTEVCDELMSRHNIYVQAINYPTVPRGEELLRI 572
ALAS1  Macaca mulatta            QMLMDAGLPVVHCPSHIIPVRVADAAKNTEVCDELMSRHNIYVQAINYPTVPRGEELLRI 572
ALAS1  Equus caballus            QMLMDAGLPVVHCPSHIIPVRVADAAKNTEVCDELMSRHNIYVQAINYPTVPRGEELLRI 572
ALAS1  Odobenus rosmarus         QMLMDAGLPVVHCPSHIIPVRVADAAKNTEVCDELMSRHNIYVQAINYPTVPRGEELLRI 639
ALAS1  Lontra canadensis         QMLMDAGLPVVHCPSHIIPVRVADAAKNTEVCDELMSRHNIYVQAINYPTVPRGEELLRI 572
ALAS1  Hyaena hyaena             QMLMDAGLPVVHCPSHIIPVRVADAAKNTEVCDELMSRHNIYVQAINYPTVPRGEELLRI 572
ALAS1  Acinonyx jubatus          QMLMDAGLPVVHCPSHIIPVRVANAAKNTEVCDELMSRHNIYVQAINYPTVPRGEELLRI 572
ALAS1  Panthera tigris           QMLMDAGLPVVHCPSHIIPVRVADAAKNTEVCDELMSRHNIYVQAINYPTVPRGEELLRI 572
ALAS1  Panthera pardus           QMLMDAGLPVVHCPSHIIPVRVADAAKNTEVCDELMSRHNIYVQAINYPTVPRGEELLRI 572
ALAS1  Lynx canadensis           QMLMDAGLPVVHCPSHIIPVRVADAAKNTEVCDELMSRHNIYVQAINYPTVPRGEELLRI 572
```

```
ALAS1 Puma yagouaroundi          QMLMDAGLPVVHCPSHIIPVRVADAAKNTEVCDELMSRHNIYVQAINYPTVPRGEELLRI 572
ALAS1 Puma concolor              QMLMDAGLPVVHCPSHIIPVRVADAAKNTEVCDELMSRHNIYVQAINYPTVPRGEELLRI 572
ALAS1 Felis catus                QMLMDAGLPVVHCPSHIIPVRVADAAKNTEVCDELMSRHNIYVQAINYPTVPRGEELLRI 572
ALAS1 Ailuropoda melanoleuca     QMLMDAGLPVVHCPSHIIPVRVADAAKNTEVCDELMSRHNIYVQAINYPTVPRGEELLRI 572
ALAS1 Canis lupus dingo          QMLMDAGLPVVHCPSHIIPVRVANAAKNTEVCDELMSRHNIYVQAINYPTVPRGEELLRI 631
ALAS2 Cavia porcellus            QLLMDRGLPVIPCLSHIIPIRVGDAALNSKICDLLLSKHGIYVQAINYPTVPRGEELLRL 518
ALAS2 Chinchilla lanigera        QLLMDRGLPVIPCPSHIIPIRVGNAALNSKVCDLLLSKHGIYVQAINYPTVPRGEELLRL 544
ALAS2 Rattus norvegicus          QLLMDRGFPVIPCPSHIIPIRVGNAALNSKICDLLLAKHSIYVQAINYPTVPRGEELLRL 524
ALAS2 Mus musculus               QLLMDRGFPVIPCPSHIIPIRVGNAALNSKICDLLLSKHSIYVQAINYPTVPRGEELLRL 518
ALAS2 Nannospalax galili         QLLMDRGLPVIPCPSHIIPIRVGDAALNSKICDLLLSKHNIYVQAINYPTVPRGEELLRL 517
ALAS2 Panthera tigris            QLLMDRGLPVIPCPSHIIPIRVGDAALNSKICDLLLSKHGIYVQAINYPTVPRGEELLRL 574
ALAS2 Panthera pardus            QLLMDRGLPVIPCPSHIIPIRVGDAALNSKICDLLLSKHGIYVQAINYPTVPRGEELLRL 518
ALAS2 Puma concolor              QLLMDRGLPVIPCPSHIIPIRVGDAALNSKICDLLLSKHGIYVQAINYPTVPRGEELLRL 518
ALAS2 Lynx canadensis            QLLMDKGLPVIPCPSHIIPIRVGDAALNSKICDFLLSKHGIYVQAINYPTVPRGEELLRL 518
ALAS2 Puma yagouaroundi          QLLMDRGLPVIPCPSHIIPIRVGDAALNSKICDLLLSKHGIYVQAINYPTVPRGEELLRL 518
ALAS2 Acinonyx jubatus           QLLMDRGLPVIPCPSHIIPIRVGDAALNSKICDLLLSKHGIYVQAINYPTVPRGEELLRL 518
ALAS2 Felis catus                QLLMDRGLPVIPCPSHIIPIRVGDAALNSKICDLLLSKHGIYVQAINYPTVPRGEELLRL 518
ALAS2 Lontra canadensis          QLLMDRGLPVIPCPSHIIPIRVGDAALNSKICDLLLSKHGIYVQAINYPTVPRGEELLRL 517
ALAS2 Canis lupus dingo          QLLMDRGLPVIPCPSHIIPIRVGDAALNSKICDLLLSKHGIYVQAINYPTVPRGEELLRL 518
ALAS2 Ailuropoda melanoleuca     QLLMDRGLPVIPCPSHIIPIRVGDAALNSKICDLLLSKHGIYVQAINYPTVPRGEELLRL 518
ALAS2 Odobenus rosmarus          QLLMDRSLPVIPCPSHIIPIRVGDAALNSKICDLLLSKHGIYVQAINYPTVPRGEELLRL 518
ALAS2 Castor canadensis          QLLMDKGLPVIPCPSHIIPIRVGDAALNSKICDLLLSKHSIYVQAINYPTVPRGEELLRL 518
ALAS2 Ochotona curzoniae         QLLMDRGLPVIPCPSHIIPIRVGDAALNSKICDLLLSKHGIYVQAINYPTVPRGEELLRL 518
ALAS2 Oryctolagus cuniculus      QLLMDRGLPVIPCPSHIIPIRVGDAALNSKICDLLLSKHGIYVQAINYPTVPRGEELLRL 518
ALAS2 Homo sapiens               QLLMDRGLPVIPCPSHIIPIRVGNAALNSKLCDLLLSKHGIYVQAINYPTVPRGEELLRL 518
ALAS2 Pan troglodytes            QLLMDRGLPVIPCPSHIIPIRVGNAALNSKLCDLLLSKHGIYVQAINYPTVPRGEELLRL 518
ALAS2 Equus caballus             QLLMDRGLPVIPCPSHIIPIRVGDAALNSKICDLLLSKHGIYVQAINYPTVPRGEELLRL 518
ALAS2 Bos taurus                 QLLMDRGLPVIPCPSHIIPIRVGDAMLNTRICDLLLSKYGIYVQAINYPTVPRGEELLRL 518
ALAS2 Delphinapterus leucas      QLLMDRGLPVIPCPSHIIPIRVGDAALNSRICDLLLSKHGIYVQAINYPTVPRGEELLRL 550
ALAS2 Sarcophilus harrisii       QLLMDKGLPVIACPSHIIPIRVGDAALNSRVCDLLISKYNIYVQAINYPTVPRGEELLRL 573
ALAS2 Phascolarctos cinereus     QLLLDKGFPVIACPSHIIPIRVGDAALNSRICDLLISKHNIYVQAINYPTVPRGEELLRL 519
ALAS2 Vombatus ursinus           QLLMDKGFPVIACPSHIIPIRVGDAALNSQICDLLISKHNIYVQAINYPTVPRGEELLRL 519
                                 *:*:*  .:**: ********:**.:*   *: :*: *::::.*********** *******:

ALAS1 Monodelphis domestica      APTPHHTPQMMNYFLENLLDTWKLVGLDLKPHSSAECNFCRRPLHFEVMSERERAYFQGM 631
ALAS1 Sarcophilus harrisii       APTPHHTPQMMNYFLENLLDTWKSVGLDLKPHSSAECNFCRRPLHFEVMSERERAYFQGM 631
ALAS1 Phascolarctos cinereus     APTPHHTPQMMNYFLENLLETWKVVGLDLKPHSSAECNFCRRPLHFEVMSEREKAYFQGL 631
ALAS1 Vombatus ursinus           APTPHHTPQMMNYFLENLLETWKFVGLDLKPHSSAECNFCRRPLHFEVMSEREKAYFQGM 631
ALAS1 Bos taurus                 APTPHHTPQMMSYFVDNLLATWKRVGLELKPHSSAECNFCRRPLHFEMMSEREKSYFSGM 693
ALAS1 Meriones unguiculatus      APTPHHTPQMMSFFLEKLLVTWKRVGLDLKPHSSAECNFCRRPLHFEVMSEREKAYFSGM 634
ALAS1 Rattus norvegicus          APTPHHTPQMMNFFLEKLLLTWKRVGLELKPHSSAECNFCRRPLHFEVMSEREKAYFSGM 634
ALAS1 Mus musculus               APTPHHTPQMMNFFVEKLLVTWKRVGLELKPHSSAECNFCRRPLHFEVMSEREKAYFSGM 634
ALAS1 Ochotona curzoniae         APTPHHTPQMMSYFLEKLLATWKQVGLDLKPHSSAECNFCRRPLHFEVMSEREKSYFSGL 680
ALAS1 Oryctolagus cuniculus      APTPHHTPQMMSYFLENLLATWKQVGLDLKPHSSAECNFCRRPLHFEVMSEREKSYFSGM 687
ALAS1 Cavia porcellus            APTPHHTPQMMSYFLENLLTTWKQVGLELKPHSSAECNFCRRPLHFEVMSEREKAYFSGL 632
ALAS1 Chinchilla lanigera        APTPHHTPQMMSYFLENLLATWKQVGLDLKPHSSAECNFCRRPLHFEVMSERERAYFSGM 632
ALAS1 Nannospalax galili         APTPHHTPQMMNFFLEKLLSTWKRVGLGLKPHSSAECNFCRRPLHFEVMSEREKAYFSGM 632
ALAS1 Castor canadensis          APTPHHTPQMMKYFLENLLATWKRVGLELKPHSSAECNFCRRPLHFEVMSERERAYFTGM 632
ALAS1 Delphinapterus leucas      APTPHHTPQMMNYFVENLLATWKRVGLELKPHSSAECNFCRRPLHFEVMSEREKSYFFGM 656
ALAS1 Homo sapiens               APTPHHTPQMMNYFLENLLVTWKQVGLELKPHSSAECNFCRRPLHFEVMSEREKSYFSGL 632
ALAS1 Pan troglodytes            APTPHHTPQMMNYFLENLLVTWKQVGLELKPHSSAECNFCRRPLHFEVMSEREKSYFSGL 632
ALAS1 Macaca mulatta             APTPHHTPQMMNYFLENLLVTWKQVGLELKPHSSAECNFCRRPLHFEVMSEREKSYFSGL 632
ALAS1 Equus caballus             APTPHHTPQMMNYFLENLLATWKRVGLELKPHSSAECNFCRRPLHFEVMSEREKSYFSGM 632
ALAS1 Odobenus rosmarus          APTPHHTPQMMNYFLENLLATWKRVGLELKPHSSAECNFCRRPLHFEVMSEREKSYFSGM 699
ALAS1 Lontra canadensis          APTPHHTPQMMSYFLENLLATWKRVGLELKPHSSAECNFCRRPLHFEVMSEREKSYFSGM 632
ALAS1 Hyaena hyaena              APTPHHTPQMMNYFLENLLATWKRVGLELKPHSSAECNFCRRPLHFEVMSEREKSYFSGM 632
ALAS1 Acinonyx jubatus           APTPHHTPQMMNYFLENLLATWKRVGLELKPHSSAECNFCRRPLHFEVMSEREKSYFSGM 632
ALAS1 Panthera tigris            APTPHHTPQMMNYFLENLLATWKRVGLELKPHSSAECNFCRRPLHFEVMSEREKSYFSGM 632
ALAS1 Panthera pardus            APTPHHTPQMMNYFLENLLATWKRVGLELKPHSSAECNFCRRPLHFEVMSEREKSYFSGM 632
ALAS1 Lynx canadensis            APTPHHTPQMMNYFLENLLATWKRVGLELKPHSSAECNFCRRPLHFEVMSEREKSYFSGM 632
ALAS1 Puma yagouaroundi          APTPHHTPQMMNYFLENLLATWKRVGLELKPHSSAECNFCRRPLHFEVMSEREKSYFSGM 632
ALAS1 Puma concolor              APTPHHTPQMMNYFLENLLATWKRVGLELKPHSSAECNFCRRPLHFEVMSEREKSYFSGM 632
ALAS1 Felis catus                APTPHHTPQMMNYFLENLLATWKRVGLELKPHSSAECNFCRRPLHFEVMSEREKSYFSGM 632
ALAS1 Ailuropoda melanoleuca     APTPHHTPQMMNYFLENLLATWKRVGLELKPHSSAECNFCRRPLHFEVMSEREKSYFSGM 632
ALAS1 Canis lupus dingo          APTPHHTPQMMNYFLENLLATWKRVGLELKPHSSAECNFCRRPLHFEVMSEREKSYFSGM 691
ALAS2 Cavia porcellus            APSPHHSPQMMEDFVGKLLVAWTEVGLPLQDVSVAACNFCHRPVHFELMSEWERSYFGNM 578
ALAS2 Chinchilla lanigera        APSPHHSPQMMEDFVGKLLVAWTEVGLPLQDVSVAACNFCHRPVHFELMSEWERSYFGNM 604
ALAS2 Rattus norvegicus          APSPHHSPQMMENFVEKLLLAWTEVGLPLQDVSVAACNFCRRPVHFELMSEWERSYFGNM 584
ALAS2 Mus musculus               APSPHHSPQMMENFVEKLLLAWTEVGLPLQDVSVAACNFCHRPVHFELMSEWERSYFGNM 578
ALAS2 Nannospalax galili         APSPHHTPQMMENFVEKLLLTWTEVGLPLQDVSVAACNFCQRPVHFELMSEWERSYFGNM 577
ALAS2 Panthera tigris            APSPHHSPQMMDDFVEKLLVAWTEVGLPLQDVSMAACNFCHRPVHFELMSEWERSYFGNM 634
```

```
ALAS2 Panthera pardus             APSPHHSPQMMDDFVEKLLVAWTEVGLPLQDVSMAACNFCHRPVHFELMSEWERSYFGNM 578
ALAS2 Puma concolor               APSPHHSPQMMDDFVEKLLVAWTEVGLPLQDVSMAACNFCHRPVHFELMSEWERSYFGNM 578
ALAS2 Lynx canadensis             APSPHHSPQMMDDFVEKLLVAWTEVGLPLQDVSMAACNFCHRPVHFELMSEWERSYFGNM 578
ALAS2 Puma yagouaroundi           APSPHHSPQMMDDFVEKLLVAWTEVGLPLQDVSMAACNFCHRPVHFELMSEWERSYFGNM 578
ALAS2 Acinonyx jubatus            APSPHHSPQMMDDFVEKLLVAWTEVGLPLQDVSMAACNFCHRPVHFELMSEWERSYFGNM 578
ALAS2 Felis catus                 APSPHHSPQMMDDFVEKLLVAWTEVGLPLQDVSMAACNFCHRPVHFELMSEWERSYFGNM 578
ALAS2 Lontra canadensis           APSPHHSPQMMEDFVQKLLVAWSEVGLPLQDVSMAACNFCHRPVHFELMSEWERSYFGNM 577
ALAS2 Canis lupus dingo           APSPHHSPQMMEDFVQKLLVAWTEVGLPLQDVSMAACNFCHRPVHFELMSEWERSYFGNM 578
ALAS2 Ailuropoda melanoleuca      APSPHHSPQMMEDFVQKLLVAWTEVGLPLQDVSMAACNFCHRPVHFELMSEWERSYFGNM 578
ALAS2 Odobenus rosmarus           APSPHHSPQMMEDFVQKLLVAWTEVELPLQDVSMAACNFCHRPVHFELMSEWERSYFGNM 578
ALAS2 Castor canadensis           APSPHHSPQMMENFVEKLLVTWTEVGLPLQDVSVAACNFCHRPVHFELMSEWERSYFGNM 578
ALAS2 Ochotona curzoniae          APSPHHSPQMMEDFVEKLLAAWTEVGLPLQDLSVAACNFCHRPVHFELMSEWERSYFGNM 578
ALAS2 Oryctolagus cuniculus       APSPHHSPQMMEDFVEKLLVAWNEVGLPLQDVSVAACNFCHRPVHFELMSEWERSYFGNM 578
ALAS2 Homo sapiens                APSPHHSPQMMEDFVEKLLLAWTAVGLPLQDVSVAACNFCRRPVHFELMSEWERSYFGNM 578
ALAS2 Pan troglodytes             APSPHHSPQMMEDFVEKLLLAWTAVGLPLQDVSVAACNFCRRPVHFELMSEWERSYFGNM 578
ALAS2 Equus caballus              APSPHHSPQMMEDFVEKLLEAWTEVGLPLQDVSMAACNFCRRPVHFELMSEWERSYFGNM 578
ALAS2 Bos taurus                  APSPHHSPQMMEDFVEKLLEAWTEVGLPLQDISIAACNFCRRPVHFELMSEWERSYFGNM 578
ALAS2 Delphinapterus leucas       APSPHHSPQMMEDFVEKLLAAWTEVGLPLQDVSIAACNFCRRPVHFELMSEWERSYFGNM 610
ALAS2 Sarcophilus harrisii        APSPHHTPQMMDYFVDKLLEAWIEVGLPLQDIPSAACNFCHRPVHFDLMSEWERSYFGNM 633
ALAS2 Phascolarctos cinereus      APSPHHTPQMMDYFVEKLLEAWAEVGLPLQDVSSAACNFCHRPVHFDLMSEWERSYFGNM 579
ALAS2 Vombatus ursinus            APSPHHTPQMMDYFVGKLLETWAEVGLPLQDVSSAACNFCHRPVHFDLMSEWERSYFGNM 579
                                  **:***:****. *: :** :*  * * *:    * ****:**:**::*** *::** .:
                                                                      C-terminal extension

ALAS1 Monodelphis domestica       SKLVSVSA-          639
ALAS1 Sarcophilus harrisii        SKLVSVSA-          639
ALAS1 Phascolarctos cinereus      SKLVSVSA-          639
ALAS1 Vombatus ursinus            SKLVSVSA-          639
ALAS1 Bos taurus                  SKLVSAQA-          701
ALAS1 Meriones unguiculatus       SKMVSAQA-          642
ALAS1 Rattus norvegicus           SKMVSAQA-          642
ALAS1 Mus musculus                SKMVSAQA-          642
ALAS1 Ochotona curzoniae          SKLVSAQA-          688
ALAS1 Oryctolagus cuniculus       SKLVSAQA-          695
ALAS1 Cavia porcellus             SKLVSAQA-          640
ALAS1 Chinchilla lanigera         SRLVSAQA-          640
ALAS1 Nannospalax galili          SKMVSAQA-          640
ALAS1 Castor canadensis           SKMVSARA-          640
ALAS1 Delphinapterus leucas       SKLVSAQA-          664
ALAS1 Homo sapiens                SKLVSAQA-          640
ALAS1 Pan troglodytes             SKLVSAQA-          640
ALAS1 Macaca mulatta              SKLVSAQA-          640
ALAS1 Equus caballus              SKLVSAQA-          640
ALAS1 Odobenus rosmarus           SKLVSARA-          707
ALAS1 Lontra canadensis           SKLVSAQA-          640
ALAS1 Hyaena hyaena               SKLVSAQA-          640
ALAS1 Acinonyx jubatus            SKLVSAQA-          640
ALAS1 Panthera tigris             SKLVSAQA-          640
ALAS1 Panthera pardus             SKLVSAQA-          640
ALAS1 Lynx canadensis             SKLVSAQA-          640
ALAS1 Puma yagouaroundi           SKLVSAQA-          640
ALAS1 Puma concolor               SKLVSAQA-          640
ALAS1 Felis catus                 SKLVSAQA-          640
ALAS1 Ailuropoda melanoleuca      SKLVSAQA-          640
ALAS1 Canis lupus dingo           SKLVSAQA-          699
ALAS2 Cavia porcellus             GPQYVTTYA          587
ALAS2 Chinchilla lanigera         GPQYVTTYA          613
ALAS2 Rattus norvegicus           GPQYVTTYA          593
ALAS2 Mus musculus                GPQYVTTYA          587
ALAS2 Nannospalax galili          GPQYVTTYA          586
ALAS2 Panthera tigris             GPQYVTTYA          643
ALAS2 Panthera pardus             GPQYVTTYA          587
ALAS2 Puma concolor               GPQYVTTYA          587
ALAS2 Lynx canadensis             GPQYVTTYA          587
ALAS2 Puma yagouaroundi           GPQYVTTYA          587
ALAS2 Acinonyx jubatus            GPQYVTTYA          587
ALAS2 Felis catus                 GPQYVTTYA          587
ALAS2 Lontra canadensis           GPQYVTTYA          586
ALAS2 Canis lupus dingo           GPQYVTTYA          587
ALAS2 Ailuropoda melanoleuca      GPQYVTTYA          587
ALAS2 Odobenus rosmarus           GPQYVTTYA          587
ALAS2 Castor canadensis           GPQYVTTYA          587
```

```
ALAS2 Ochotona curzoniae      GPQYVTTYA       587
ALAS2 Oryctolagus cuniculus   GPQYVTTYA       587
ALAS2 Homo sapiens            GPQYVTTYA       587
ALAS2 Pan troglodytes         GPQYVTTYA       587
ALAS2 Equus caballus          GPQYVTTYA       587
ALAS2 Bos taurus              GPQYVTTYA       587
ALAS2 Delphinapterus leucas   GPQYVTTYA       619
ALAS2 Sarcophilus harrisii    GPQYVTVFA       642
ALAS2 Phascolarctos cinereus  GPQYVTVFA       588
ALAS2 Vombatus ursinus        GPQYVTVFA       588
                              .    .
```

Supplementary Figure 2. Clustal alignment of ALAS1 and ALAS2 sequences from a variety of mammals reveals key regions of structural conservation and divergence between the isozymes. Purple highlights indicate residues conserved in both ALAS1 and ALAS2, while green and yellow indicate divergences. The five HRMs are highlighted in red, the active site lysine is highlighted blue, the active site loop is highlighted in silver, and the C-terminal CXXC motif is highlighted in gold.

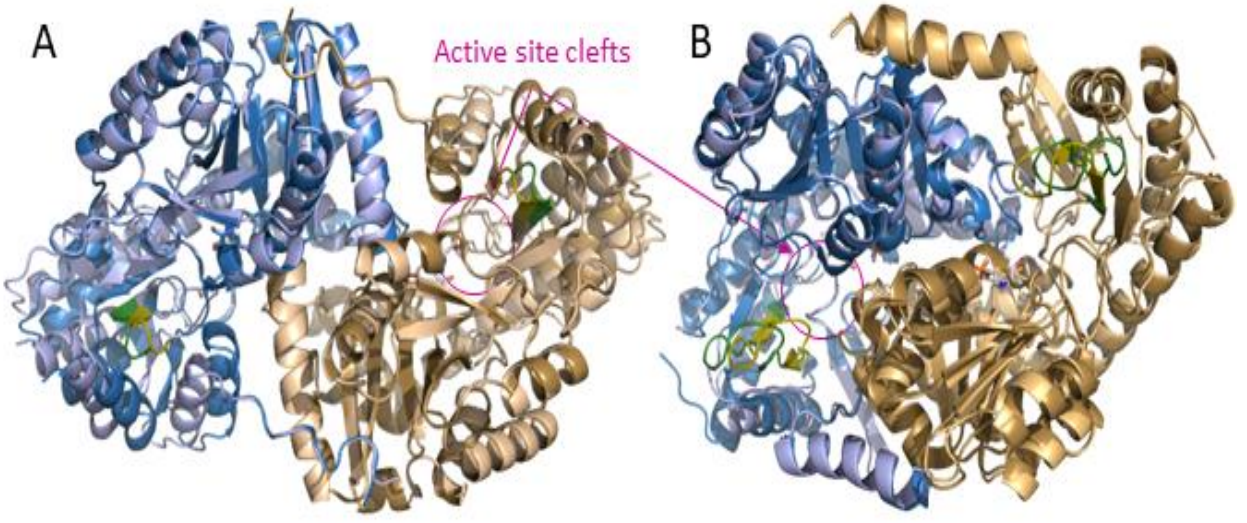

Supplementary Figure 3. Comparison of the catalytic core tertiary structures of ALAS and AATase. A. The dimeric structures of open and closed conformations of chicken mitochondrial AATase (PDB codes: 9AAT and 1AMA, respectively) are aligned. The individual subunits are in shades of blue and brown, with the darker shades representing the closed conformation, and the lighter shades representing the open conformation. Additionally, the loop regions corresponding to the "open" and "closed" conformations discussed in the text are depicted in green and gold, respectively. The PLP cofactors associated with each subunit can be partially seen in white-carbon stick representation near the center of the structures at the base of the active site clefts. **B**. Analogous structures of ALAS in the open and closed conformations (PDB codes: 6HRH and 2BWN, respectively) are depicted in the same colors for direct comparison.

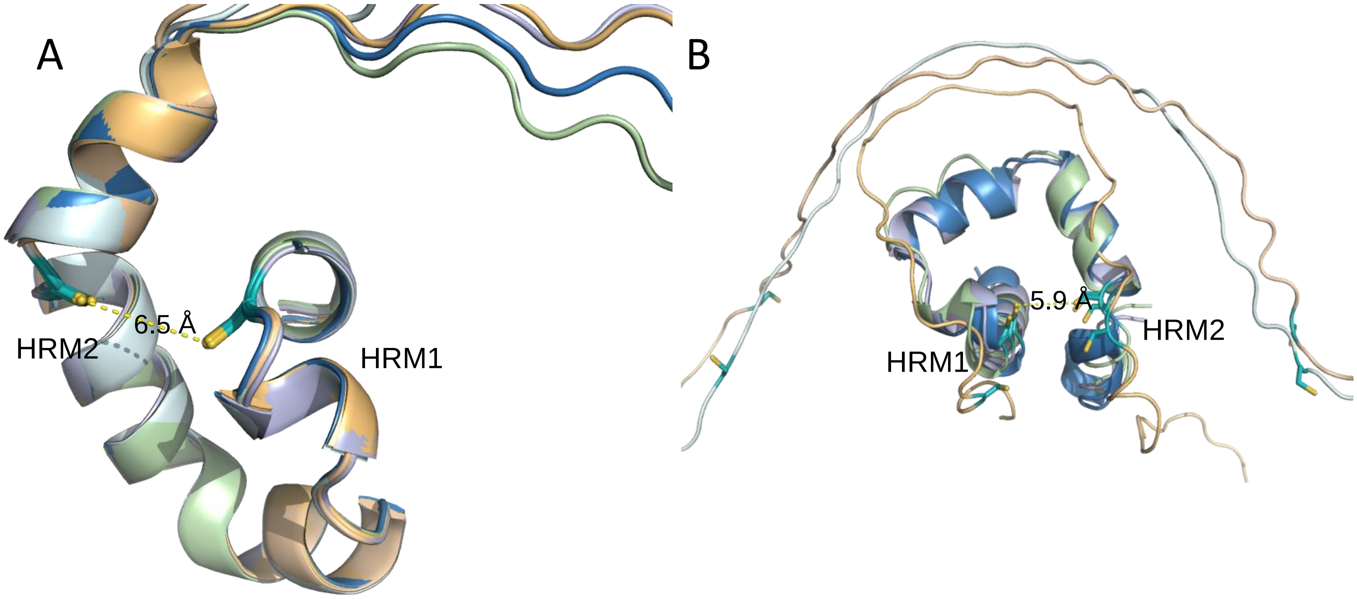

Supplementary Figure 4. Alignments of the AlphaFold-predicted structures for import presequences of mammalian ALAS1 (panel A) and ALAS2 (panel B). Alignments were conducted in Pymol using AlphaFold-predicted structures for ALAS1 from human (UniProt accession # P13196), orangutan (UniProt accession Q5R9R9), bovine (UniProt accession A6QLI6), beluga whale (UniProt accession Q9XS79), Mouse (UniProt accession Q8VC19), and rat (UniProt accession P13195), and for ALAS2 from human (UniProt accession P22557), orangutan (UniProt accession Q5R557), bovine (UniProt accession Q3ZC31), beluga whale (UniProt accession Q9XT75), mouse (UniProt accession P08680), and rat (UniProt accession Q63147). The relative positioning of HRM1 and HRM2 suggest that they axially coordinate heme to form a six-coordinate complex. The extended conformations of bovine ALAS2 (light blue), orangutan ALAS2 (upper beige), and beluga whale ALAS2 (lower beige) are likely not erroneous and, instead, the collective structures can be taken as an indication that the ALAS2 presequence has a greater deal of intrinsic disorder than the ALAS1 presequence[52].

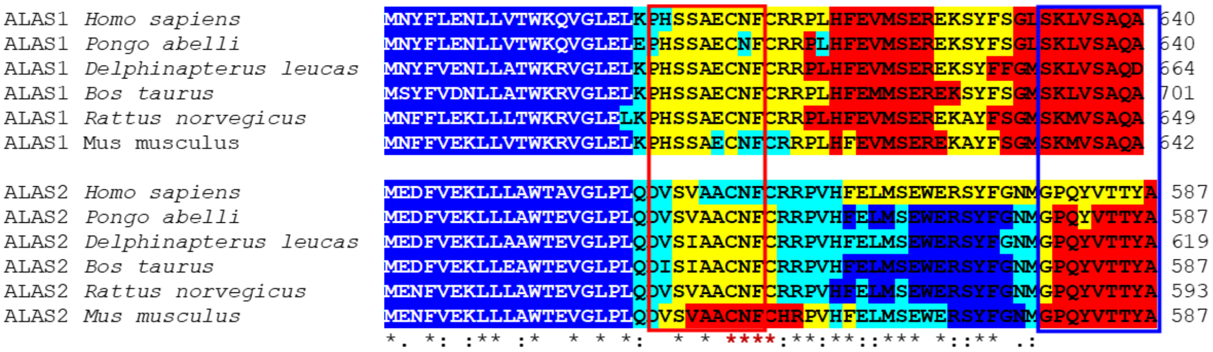

Supplementary Figure 5. pLDDT scores for ALAS1 and ALAS2 C-terminal extensions indicate substantially greater conformational heterogeneity than for the catalytic core. Highlights indicate the structural model confidence as follows: dark blue and white font, very high (pLDDT > 90); light blue, good (70 > pLDDT > 90); yellow, low (50 > pLDDT > 70); red, very low (pLDDT < 50). Residues boxed in red and blue are missing from the human ALAS2 crystal structure. The C-terminal extension starts three residues before the CXXC motif postulated to have a redox function and denoted here by red asterisks. Note that the only ALAS2 structure with a confident score for structural homogeneity about the CXXC motif is for the human ALAS2 structure, which predicts a conformation distinct from the other Alphafold structures as well as the human ALAS2 crystal structure.
